# Gestational weight gain outside the 2009 Institute of Medicine recommendations: novel psychological and behavioural factors associated with inadequate or excess weight gain in a prospective cohort study

**DOI:** 10.1186/s12884-021-03555-5

**Published:** 2021-01-21

**Authors:** Yu Yang Feng, Zhijie Michael Yu, Sherry van Blyderveen, Louis Schmidt, Wendy Sword, Meredith Vanstone, Anne Biringer, Helen McDonald, Joseph Beyene, Sarah Diana McDonald

**Affiliations:** 1 Department of Obstetrics and Gynecology, McMaster University, Hamilton, Ontario Canada; 2 Eating Disorders Program at Homewood Health Centre, Guelph, Ontario Canada; 3 Department of Psychology, Neuroscience & Behaviour, McMaster University, Hamilton, Ontario Canada; 4 School of Nursing, McMaster University, Hamilton, Ontario Canada; 5 Department of Family Medicine, McMaster University, Hamilton, Ontario Canada; 6 Ray D. Wolfe Department of Family Medicine, Mount Sinai Hospital, Toronto, Ontario Canada; 7 Midwifery Education Program, McMaster University, Hamilton, Ontario Canada; 8 Department of Health Research Methods, Evidence & Impact, McMaster University, Hamilton, Ontario Canada; 9 Department of Radiology, McMaster University, Hamilton, Ontario Canada; 10 Division of Maternal-Fetal Medicine, Department of Obstetrics and Gynecology, McMaster University, 1280 Main Street West, room 3N52B, Hamilton, Ontario L8S 4K1 Canada

**Keywords:** Inadequate gestational weight gain, Excess gestational weight gain, Body mass index, Psychology, Behaviour, Prospective cohort study

## Abstract

**Background:**

Previous studies have noted traditional physical, demographic, and obstetrical predictors of inadequate or excess gestational weight gain, but the roles of psychological and behavioral factors are not well established. Few interventions targeting traditional factors of gestational weight gain have been successful, necessitating exploration of new domains. The objective of this study was to identify novel psychological and behavioral factors, along with physical, demographic, and obstetrical factors, associated with gestational weight gain that is discordant with the 2009 Institute of Medicine guidelines (inadequate or excess gain).

**Methods:**

We recruited English-speaking women with a live singleton fetus at 8 to 20 weeks of gestation who received antenatal care from 12 obstetrical, family medicine, and midwifery clinics. A questionnaire was used to collect information related to demographic, physical, obstetrical, psychological, and behavioural factors anticipated to be related to weight gain. The association between these factors and total gestational weight gain, classified as inadequate, appropriate, and excess, was examined using stepwise multinomial logistic regression.

**Results:**

Our study population comprised 970 women whose baseline data were obtained at a mean of 14.8 weeks of gestation ±3.4 weeks (standard deviation). Inadequate gestational weight gain was associated with obesity, planned gestational weight gain (below the guidelines or not reported), anxiety, and eating sensibly when with others but overeating when alone, while protective factors were frequent pregnancy-related food cravings and preferring an overweight or obese body size image. Excess gestational weight gain was associated with pre-pregnancy overweight or obese body mass index, planned gestational weight gain (above guidelines), frequent eating in front of a screen, and eating sensibly when with others but overeating when alone, while a protective factor was being underweight pre-pregnancy.

**Conclusions:**

In addition to commonly studied predictors, this study identified psychological and behavioral factors associated with inadequate or excess gestational weight gain. Factors common to both inadequate and excessive gestational weight gain were also identified, emphasizing the multidimensional nature of the contributors to guideline-discordant weight gain.

**Supplementary Information:**

The online version contains supplementary material available at 10.1186/s12884-021-03555-5.

## Background

Gestational weight gain (GWG) outside of the recommendations [1] (inadequate or excess GWG) is associated with an increased risk of adverse outcomes in mothers and infants [2–5]. Inadequate GWG is associated with a 70% increase in the odds of preterm birth [5], the leading cause of neonatal morbidity and mortality [6], as well as increases in low birthweight, small-for-gestational-age infants, and failure to initiate breastfeeding [7, 8]. Excess GWG is associated with Caesarean birth, high birthweight, large-for-gestational-age infants, and postpartum weight retention [7, 8].

Guideline-discordant GWG is a global concern, as in many regions the proportion of women whose GWG is outside of the recommendations is substantial. A recent meta-analysis of over 1 million women reported that in the United States, Europe, and Asia, rates of inadequate GWG were 21, 18, and 31%, respectively, and those of excess GWG were 51, 51, and 37%, respectively [7]. Rates of inadequate and excess GWG in Canada were reported by a study conducted in 2009–2012 to be similar at 18 and 49%, respectively [9]. As guideline-discordant GWG is a potentially modifiable risk factor for adverse perinatal outcomes, it is important to understand predictors to be able to minimize its incidence.

The United States Institute of Medicine (IOM) adopted the recommendations on GWG in 2009 [1]. The recommendations were adapted by Health Canada [10] and several other countries [11]. The 2009 IOM recommendations vary based on prepregnancy body mass index (BMI). The recommended total weight gains for women classified as underweight (BMI < 18.5 kg/m^2^), normal weight (18.5 ≤ BMI < 25 kg/m^2^), overweight (25 ≤ BMI < 30 kg/m^2^), and obese (BMI ≥30 kg/m^2^) are 12.5–18 kg (28–40 lbs.), 11.5–16 kg (25–35 lbs.), 7–11.5 kg (15–25 lbs.), and 5–9 kg (11–20 lbs.), respectively [1, 10].

While traditional physical, demographic, and obstetrical factors for guideline-discordant GWG have been explored [12–17], meta-analyses have identified few effective interventions directed toward these factors. A broadening of the approach to unexplored domains such as psychological and behavioral factors has been suggested [15, 16], and our prospective cohort study investigated such novel and potentially modifiable predictors of weight gain. Our selection of psychological and behavioural factors to be studied was informed by a systematic review [18]. Behavioural factors included diet, eating in front of a screen, sleep, physical activity, pregnancy-related nausea, and food cravings, as well as the means to cope with nausea and food cravings. Psychological domains included cognition, affect, and personality. Our previous analysis [19] of this dataset formed the first validated predictive model for excess GWG (compared to not excess gain, combining gain within or below guidelines as the reference). This study extends our previous research by investigating the impact of these novel factors on inadequate GWG as well as excess GWG. It is important to study both extremes of guideline-discordant GWG to account for the possibility that protective factors against inadequate GWG may increase excess GWG or vice versa. Thus, this study aimed to expand the literature on both inadequate and excess GWG, as defined by the 2009 IOM recommendations, by investigating the impact of novel predictors such as personality, cognition, affect, and behaviour, in addition to other psychological, behavioral, physical, demographic, and obstetrical factors to develop a more holistic understanding of guideline-discordant GWG.

## Methods

### Study sample

In the prospective cohort study, we recruited 970 women who received antenatal care from obstetricians, family practitioners, and midwives at 12 clinics throughout the five regions of Ontario from 2015 to 2017. The study methods of the cohort were previously described [19]. Women were eligible for the study if they were English-speaking with a live singleton fetus from 8 weeks and 0 days up to 20 weeks and 6 days gestation. There were 1050 women who were recruited early in their pregnancy and completed the baseline data questionnaire. We followed the women to the end of their pregnancies and extracted the outcome data from their antenatal records. We excluded 80 women from the analysis based on the following criteria: 1) pregnancy with twins or higher-order multiples; 2) a fetus with a known lethal anomaly, a fetal demise, or a termination of pregnancy after enrollment; 3) maternal pathological conditions that affect weight gain; or 4) missing antenatal records for study outcome assessments [19]. The Hamilton Integrated Research Ethics Board (REB #13–021) and local REB committees reviewed and approved the study before its initiation. We obtained informed consent from all participants prior to data collection.

### Development of the questionnaire

Six content experts (an obstetrician, a clinical psychologist, a research personality psychologist, a perinatal nurse, a midwife, and a family physician) developed the questionnaire on sociodemographic, psychological, and behavioural factors related to weight gain, which has been published previously [19].

From the questionnaires, we obtained data on marital status, education level, and annual household income (Additional file 1). We classified women as never having smoked, currently smoking, or having quit smoking. We defined chronic health conditions, depression, and anxiety as any such condition diagnosed by a physician.

We calculated prepregnancy BMI as weight in kilograms divided by height in meters squared. We classified prepregnancy BMI as underweight (< 18.5 kg/m^2^), normal weight (18.5–24.9 kg/m^2^), overweight (25–29.9 kg/m^2^), and obese (≥30 kg/m^2^) according to the IOM [1] and World Health Organization criteria [20]. We assessed planned weight gain with the question, “How much total weight do you plan to gain during this pregnancy?” and classified the responses as within, below, or above the IOM guidelines, or not reported. We asked participants to report the recommendations of their healthcare providers regarding weight gain in the first trimester. Such recommendations were categorized as none, within the IOM guidelines, and outside the IOM guidelines. Healthcare providers’ recommendations on total weight gain were also classified as within, below, or above the IOM guidelines, none, or not reported/I can’t remember [1]. We obtained total GWG from the antenatal record by subtracting prepregnancy weight from the final pregnancy weight.

The data collection and definitions of variables for health and pregnancy-related behaviours, as well as psychological factors, are detailed in the cohort’s initial publication [19]. In brief, behavioural factors included diet, eating in front of a screen, sleep, physical activity, pregnancy-related nausea, and food cravings, as well as the means to cope with nausea and food cravings. Diet was assessed with a numerous multiple-choice questions to determine the frequency of behaviours such as drinking soda/juice, eating fast food, eating fruits and vegetables, eating snack foods such as cookies and chips, and eating in the middle of the night. The frequency of eating in front of a screen was similarly assessed. Sleep and physical activity were assessed using a picture and validated scale adapted from Aadahl 2003, which was used to obtain a 24-h MET-time (metabolic equivalent of task) score [21]. Nausea and food cravings were assessed using Likert scales and the means to cope with nausea and food cravings were assessed with multiple-choice questions. Guided by our previous systematic review [18] on psychological factors and GWG and a pilot study [22], we selected validated psychological scales or subscales, or items from such scales and subscales, to assess the following psychological domains: 1) cognition (attitudes on body weight, body image, self-efficacy, weight locus of control, dietary restraint, and barriers to healthy eating); 2) affect (depression, anxiety); and 3) personality (impulse control, perfectionism, motivation, emotional suppression, and the Big Five Personality Factors [extraversion, agreeableness, conscientiousness, emotional stability, and openness]) (Additional file 2).

### Assessment of study outcomes

Our study outcome was total GWG, which was classified as inadequate, appropriate, or excessive according to the IOM recommendations [1]. Total GWG was calculated by subtracting the prepregnancy weight from the final measured weight during pregnancy. Weight and height measurements were extracted from provincial Ministry of Health Antenatal Records [23].

### Statistical analysis

We summarized descriptive statistics stratified by GWG status by calculating frequencies and proportions for categorical variables, and means and standard deviations for continuous variables (Additional file 1). We used chi-square tests and analyses of variance (ANOVA) for categorical and continuous variables, respectively, to test for significant differences between GWG statuses. We examined collinearity between variables using Spearman’s correlation. For variable pairs with bilateral Spearman’s correlation coefficients ≥ | ± 0.70|, we retained the more psychologically- and biologically-relevant variable. We then performed univariable multinomial logistic regression analyses to assess the associations between the exposure variables with the study outcome (i.e., GWG status), using appropriate weight gain as the reference group (Additional file 3). We employed stepwise multinomial logistic regression for the selection of important exposure variables related to inadequate or excess GWG. A *p*-value cutoff of < 0.10 was used for entry into the variable selection procedure, as defined by the likelihood ratio test statistic [24]. We retained statistically significant variables with a two-sided *p*-value < 0.05. Missing data were generally low, varying from 0.1 to 3.2% among the 78 exposure variables included in the study. Few variables had greater than 7% missing data, with the highest variable being family income at 9.59%. We used the fully conditional specification method to create 10 imputed data sets [25] with PROC MI in SAS [26, 27]. For variables in the stepwise regression analysis, we calculated the means of the 10 imputed values and rounded them to the nearest integers for categorical variables, and to the nearest decimal values for continuous variables. We used SAS 9.4 software (SAS Institute, Cary, North Carolina) for data management and statistical analysis.

## Results

**Fig. 1:**
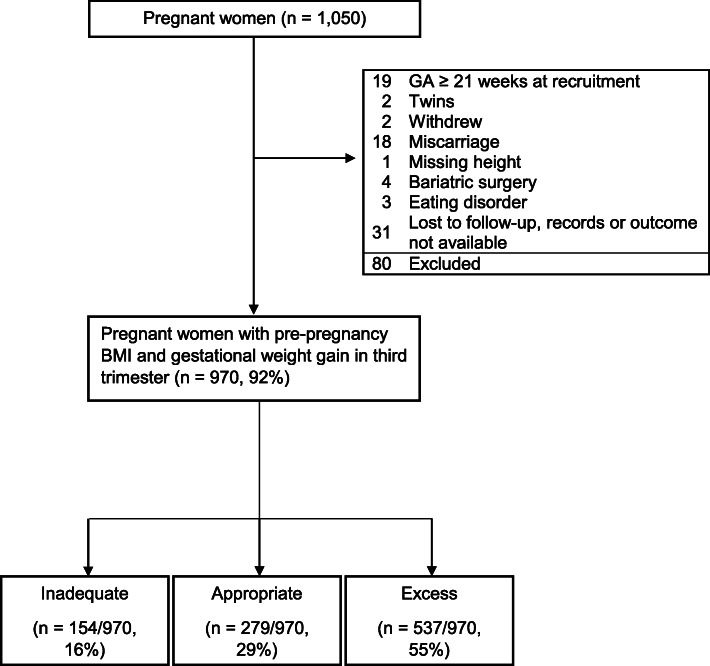
Flowchart of sample selection in a prospective cohort study on guideline-discordant gestational weight gain. Due to use of the same dataset, parts of this figure resemble data found in: McDonald SD, Yu ZM, Blyderveen S van, et al. (2020) Prediction of excess pregnancy weight gain using psychological, physical, and social predictors: A validated model in a prospective cohort study. PLOS ONE 15:e0233774. 10.1371/journal.pone.0233774

We approached 1296 women for participation in this study, of whom 1050 (81%) provided informed consent to participate. We obtained complete outcome data on 970 (92%) women, who form our study population. Their baseline data were obtained at a mean of 14.8 weeks of gestation ±3.4 weeks (standard deviation) (Fig. 1, Table 1). There were no significant differences between women with and without missing data in maternal age, gestational age at recruitment, prepregnancy BMI, or GWG outcome. The majority of women were Caucasian, had undergraduate or higher levels of education, had a household income ≥$80,000 (Canadian dollars), were married, common-law, or living with a partner, and did not smoke. The mean maternal age was 30.5 years (Table 1). Approximately 28.8% (279/970) of women gained weight within the IOM guidelines, while 15.9% (154/970) gained weight below the IOM guidelines, and 55.4% (537/970) gained weight in excess of the guidelines. Differences in exposure variables between women in each GWG group are shown in Additional file 1.

**Table 1:** Participant characteristics by weight gain level in prospective cohort study on guideline-discordant gestational weight gain

Characteristic	Inadequate weight gain *n* = 154		Appropriate weight gain *n* = 279		Excess weight gain *n* = 537		***p***-value
**Maternal age, years** [Mean (SD)]	30.8	(4.5)	30.9	(5.0)	30.2	(4.9)	0.097
**Gestational age at recruitment, weeks** [Mean (SD)]	14.8	(3.3)	14.4	(3.4)	15.1	(3.5)	0.018
**Race** [n (%)]							0.011
White	110	(71.4)	199	(71.3)	427	(79.5)	
Non-white	43	(27.9)	80	(28.7)	108	(20.1)	
NR/UKN	1	(0.6)	0	(0.0)	2	(0.4)	
**Marital status** [n (%)]							0.997
Married, common-law, or living with a partner	143	(92.9)	259	(92.8)	496	(92.4)	
Single, divorced, or widowed	11	(7.1)	20	(7.2)	39	(7.3)	
NR/UKN	0	(0.0)	0	(0.0)	2	(0.4)	
**Education** [n (%)]							0.592
Community college/technical school or lower	69	(44.8)	112	(40.1)	231	(43.0)	
Undergraduate university or higher	85	(55.2)	167	(59.9)	305	(56.8)	
NR/UKN	0	(0.0)	0	(0.0)	1	(0.2)	
**Household income** [n (%)]							0.450
< $40,000	28	(18.2)	45	(16.1)	79	(14.7)	
$40,000 – 79,999	39	(25.3)	64	(22.9)	147	(27.4)	
> =$80,000	75	(48.7)	149	(53.4)	251	(46.7)	
NR or prefer not to answer	12	(7.8)	21	(7.5)	60	(11.2)	
**Smoking** [n (%)]							0.015
None	128	(83.1)	235	(84.2)	414	(77.1)	
Before this pregnancy	12	(7.8)	27	(9.7)	85	(15.8)	
During this pregnancy	14	(9.1)	15	(5.4)	38	(7.1)	
NR/UKN	0	(0.0)	2	(0.7)	0	(0.0)	
**Chronic health conditions** [n (%)]							0.685
No	109	(70.8)	208	(74.6)	390	(72.6)	
Yes	45	(29.2)	71	(25.4)	147	(27.4)	
**Parity** [n (%)]							0.115
0	72	(46.8)	138	(49.5)	296	(55.1)	
1+	81	(52.6)	139	(49.8)	239	(44.5)	
NR/UKN	1	(0.6)	2	(0.7)	2	(0.4)	
**Prepregnancy BMI** [n (%)]							< 0.001
Underweight (BMI < 18.5 kg/m^2^)	6	(3.9)	15	(5.4)	8	(1.5)	
Normal weight (BMI 18.5–24.9 kg/m^2^)	101	(65.6)	174	(62.4)	218	(40.6)	
Overweight (BMI 25.0–29.9 kg/m^2^)	10	(6.5)	46	(16.5)	176	(32.8)	
Obese (BMI ≥30 kg/m^2^)	37	(24.0)	44	(15.8)	135	(25.1)	
**Care provider at centre of recruitment** [n (%)]							0.189
Obstetrician	38	(24.7)	74	(26.5)	106	(19.7)	
Midwife	18	(11.7)	40	(14.3)	77	(14.3)	
Family physician	98	(63.6)	165	(59.1)	354	(65.9)	

*BMI* body mass index, *NR* not reported, *UKN* unknown

Percentages may not total 100 due to rounding

The descriptive and univariable multinomial logistic regression analyses were complete case analyses

**Fig. 2:**
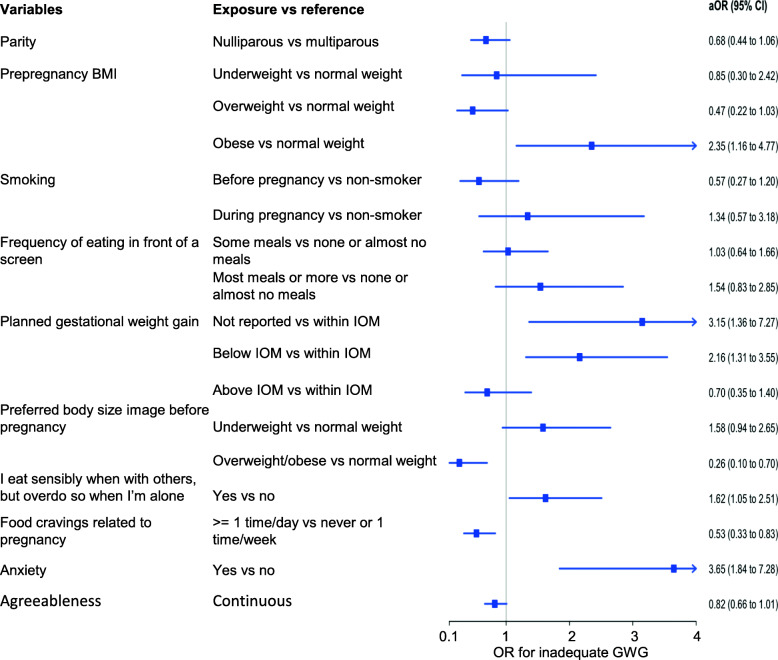
Exposure variables for inadequate weight gain in prospective cohort study on guideline-discordant gestational weight gain

**Fig. 3:**
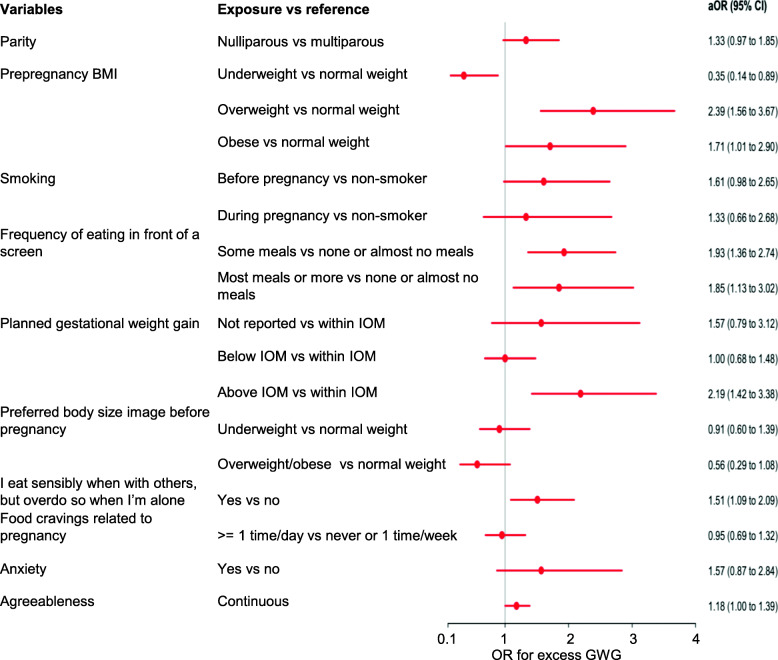
Exposure variables for excess weight gain in prospective cohort study on guideline-discordant gestational weight gain

Factors that were associated with inadequate or excess GWG in the univariable analysis (Additional file 3) were considered in a stepwise multinomial logistic regression. We identified 10 variables that were associated with inadequate or excess GWG, or both (Table 2, Fig. 2, Fig. 3): parity, prepregnancy BMI, planned GWG, preferred prepregnancy body size image, anxiety, pregnancy-related food cravings, smoking, frequency of eating in front of a screen, agreeableness, and eating sensibly when with others but overeating when alone. Although each of the 10 predictor variables was significantly different between outcome groups (GWG within, below, or above guidelines), not all exposures encompassed by those variables were statistically significant for inadequate or excess GWG individually.

**Table 2:** Exposure variables for inadequate or excess weight gain in a prospective cohort study

Selected variables/ exposure	overall ***p-value***	Selected variables/exposure	Reference	Outcome	aOR (95% CI)	***p-value*** for each category
**Parity**	0.005					
		Nulliparous	Multiparous	Inadequate GWG	0.68 (0.44 to 1.06)	0.088
				Excess GWG	1.33 (0.97 to 1.85)	0.081
**Prepregnancy BMI**	< 0.001					
		Underweight	Normal weight	Inadequate GWG	0.85 (0.30 to 2.42)	0.760
				Excess GWG	0.35 (0.14 to 0.89)	0.027
		Overweight	Normal weight	Inadequate GWG	0.47 (0.22 to 1.03)	0.058
				Excess GWG	2.39 (1.56 to 3.67)	< 0.001
		Obese	Normal weight	Inadequate GWG	2.35 (1.16 to 4.77)	0.017
				Excess GWG	1.71 (1.01 to 2.90)	0.047
**Planned gestational weight gain**	< 0.001					
		Not reported	Within guidelines	Inadequate GWG	3.15 (1.36 to 7.27)	0.007
				Excess GWG	1.57 (0.79 to 3.12)	0.202
		Below guidelines	Within guidelines	Inadequate GWG	2.16 (1.31 to 3.55)	0.003
				Excess GWG	1.00 (0.68 to 1.48)	> 0.999
		Above guidelines	Within guidelines	Inadequate GWG	0.70 (0.35 to 1.40)	0.312
				Excess GWG	2.19 (1.42 to 3.38)	< 0.001
**Preferred body size image before pregnancy**	0.013					
		Underweight	Normal weight	Inadequate GWG	1.58 (0.94 to 2.65)	0.083
				Excess GWG	0.91 (0.60 to 1.39)	0.654
		Overweight/obese	Normal weight	Inadequate GWG	0.26 (0.10 to 0.70)	0.007
				Excess GWG	0.56 (0.29 to 1.08)	0.086
**Anxiety**	0.001					
		Yes	No	Inadequate GWG	3.65 (1.84 to 7.28)	< 0.001
				Excess GWG	1.57 (0.87 to 2.84)	0.132
**Food cravings related to pregnancy**	0.014					
		≥ 1 time/day	Never or 1 time/week	Inadequate GWG	0.53 (0.33 to 0.83)	0.006
				Excess GWG	0.95 (0.69 to 1.32)	0.776
**Smoking**	0.027					
		Before pregnancy	Non-smoker	Inadequate GWG	0.57 (0.27 to 1.20)	0.136
				Excess GWG	1.61 (0.98 to 2.65)	0.059
		During pregnancy	Non-smoker	Inadequate GWG	1.34 (0.57 to 3.18)	0.502
				Excess GWG	1.33 (0.66 to 2.68)	0.420
**Frequency of eating in front of a screen**	0.001					
		Some meals	None or almost no meals	Inadequate GWG	1.03 (0.64 to 1.66)	0.898
				Excess GWG	1.93 (1.36 to 2.74)	< 0.001
		Most meals or all	None or almost no meals	Inadequate GWG	1.54 (0.83 to 2.85)	0.176
				Excess GWG	1.85 (1.13 to 3.02)	0.014
**I eat sensibly when with others, but overeat when I’m alone**	0.024					
		Yes	No	Inadequate GWG	1.62 (1.05 to 2.51)	0.030
				Excess GWG	1.51 (1.09 to 2.09)	0.013
**Agreeableness**	0.002					
		Continuous variable	–	Inadequate GWG	0.82 (0.66 to 1.01)	0.063
				Excess GWG	1.18 (1.00 to 1.39)	0.049

*BMI* body mass index, *CI* confidence interval, *GWG* gestational weight gain, *aOR* adjusted odds ratio

In the multinomial regression, inadequate GWG was associated with: obesity (adjusted odds ratio [aOR], 2.35; 95% confidence interval [CI], 1.16–4.77; Table 2, Fig. 2), planned gestational weight gain below the guidelines (aOR, 2.16; 95% CI, 1.31–3.55) or not reported (aOR, 3.15; 95% CI, 1.36–7.27), anxiety (aOR, 3.65; 95% CI, 1.84–7.28), and eating sensibly when with others but overeating when alone (aOR, 1.62; 95% CI, 1.05–2.51). Protective factors against inadequate gain were frequent pregnancy-related food cravings (aOR, 0.53; 95% CI, 0.33–0.83) and preferring an overweight or obese body size image (aOR, 0.26; 95% CI 0.10–0.70).

Excess GWG was positively associated with being overweight (aOR, 2.39; 95% CI 1.56–3.67; Table 2, Fig. 3) or obese (aOR, 1.71; 95% CI, 1.01–2.90), planned GWG above guidelines (aOR, 2.19; 95% CI, 1.42–3.38), frequent eating in front of a screen (some meals: aOR, 1.93; 95% CI, 1.36–2.74; or most meals or all: aOR, 1.85; 95% CI, 1.13–3.02), and eating sensibly when with others but overeating when alone (aOR, 1.51; 95% CI, 1.09–2.09). A protective factor against excess GWG was being underweight before pregnancy (aOR, 0.35; 95% CI, 0.14–0.89).

Although not statistically significant, nulliparity and agreeableness both approached significance in terms of being protective against inadequate GWG (aOR, 0.68; 95% CI, 0.44–1.06; and aOR, 0.82; 95% CI, 0.66–1.01, respectively) and also approached significance in terms of being associated with excess GWG (aOR, 1.33; 95% CI, 0.97–1.85; and aOR, 1.18; 95% CI, 1.00–1.39, respectively).

## Discussion

In this multicentre prospective cohort study, we identified predictors of guideline-discordant GWG (inadequate or excess GWG). For inadequate GWG, anxiety was the strongest positive predictor, whereas preferring an overweight or obese body size image was the strongest protective factor. The impact of anxiety and women’s body size preferences highlights the importance of psychological factors in addressing inadequate GWG.

Although anxiety was strongly associated with inadequate GWG in the current study, this relationship has been inconsistently demonstrated in the previous literature [28–30] and may involve complex interactions with physiological, psychological, and environmental factors such as chronic stress and anxiety, coping mechanisms, and hormonal pathways. Stress can manifest in overeating or undereating behaviours in different women [31]. Chronic stress is associated with increased intake of nutrient-dense foods and weight gain through the hypothalamic-pituitary-adrenal cortical system [31]. However, the introduction of a source of acute stress and anxiety, such as pregnancy, may lead to decreased food intake mediated by the sympathetic adrenal medullary system [31]. The balance between the chronic and acute nature of women’s stress and anxiety may be a source of variability in the relationship between anxiety and GWG.

The strongest predictors of excess GWG were related to prepregnancy BMI: overweight prepregnancy BMI conferred the greatest odds of experiencing excess GWG, whereas underweight prepregnancy BMI was the strongest protector against excess GWG. These findings reinforce the role of prepregnancy BMI in excess GWG, which may be partly related to the lesser amount of recommended weight gain for higher BMI categorizations.

Planned GWG was also a strong predictor of actual GWG, as women had greater odds of gaining below or above guidelines as they planned. Interestingly, women who did not report their planned GWG had greater odds of experiencing inadequate GWG than women who reported plans to gain weight below the IOM guidelines. While their reasons for not reporting their planned GWG are unknown, possible explanations include not having a planned GWG, not receiving counseling on GWG, not wishing to disclose it, or not remembering it. As planned GWG represents a modifiable risk factor, our study supports the importance of appropriate professional healthcare counseling on GWG. This is consistent with previous findings from Deputy and colleagues (2018) which identified that a lack of IOM-consistent advice on GWG from healthcare providers was associated with inadequate GWG, and that receiving advice that was below the recommended GWG range had a stronger association than not receiving or remembering the advice [32].

Another topic that healthcare providers can counsel women on is the frequency of eating in front of a screen, a modifiable lifestyle risk factor that was highly associated with excess GWG in our study. Distracted eating has been associated with increased dietary intake during and after meals, and “attentive-eating” principles have been suggested as an avenue for intervention [33].

Two variables were associated with both inadequate and excess GWG: having an obese pre-pregnancy BMI and eating sensibly when with others but overeating when alone. Obese pre-pregnancy BMI has been observed to be associated with increased odds of inadequate and excess GWG in previous studies in the United States [14, 34]. Potential mechanisms explaining the association between obesity and inadequate GWG may include perceptions of negative impact of weight gain on personal health, concerns about weight-related pregnancy and delivery complications, a greater likelihood others have warned them they are at high risk for complications, and difficulty of postpartum weight loss [35–37]. Mechanisms behind the association between obesity and excess GWG may include general diet behaviours, body weight regulation, and a lower recommended amount of GWG for women with obese prepregnancy BMI (thus they may believe their recommended weight gain to be the same as women with average prepregnancy BMI) [12, 14]. Eating sensibly when with others but overeating when alone was also associated with both inadequate and excess GWG. This behaviour may represent a tendency towards binge eating in some women, as women who binge eat may be more likely to do so when alone [38]. In some other women who tend towards restrained eating, the perception of “overeating when alone” may actually represent undereating. Women’s perceptions of what is considered eating sensibly or overeating may vary and be influenced by factors such as dietary beliefs and preferences, social influences by friends and family, a history of restrained eating, and advice received from healthcare providers on GWG. For example, a study by Mumford and colleagues (2008) identified that restrained eating behaviours were associated with inadequate GWG for women with underweight BMI, and excess GWG for women with normal, overweight, and obese BMI [39].

A previous Canadian cohort study identified multiparity and daily smoking during the final 3 months of pregnancy as significant predictors of inadequate GWG [12]. We observed similar trends in our study, but they did not reach our significance threshold, which may be due to the smaller sample size of women who experienced GWG below the IOM guidelines in this study.

Strengths of our study include the broad approach taken to understanding guideline-discordant GWG, including psychological, behavioral, physical, demographic, and obstetrical variables, and the inclusion of both well established factors in GWG and previously unexplored factors, such as personality. When developing the questionnaire, we used validated scales when possible and consulted experts to assess content validity. We recruited women who received prenatal care from various types of healthcare providers to increase generalizability and minimized loss to follow-up. Another strength of this study was our use of multinomial logistic regression analyses to distinguish factors related to either inadequate or excess GWG, or both. This approach enabled us to identify unique and universal factors that may be associated with IOM guideline-discordant GWG.

A limitation to our study was the low number of women who gained weight below the IOM guidelines, which reduced our statistical power in this group. The reference group in our study was women who gained weight within the IOM guidelines, however, that group was a minority due to the large proportion of women who gained weight above the IOM guidelines (55.4%). Our cohort also largely comprised women with high education and household incomes, which may limit the generalizability of the findings. Thus, future research should aim to target a more generalizable population and investigate interventions that address multiple predictors of GWG outside of the IOM recommendations, including psychological, behavioral, physical, demographic, and obstetrical factors.

## Conclusions

In this prospective cohort study on the predictors of guideline-discordant GWG, we identified psychological and behavioral factors as well as more traditional ones that were associated with inadequate or excess gestational weight gain. Factors common to both included obesity and eating sensibly when with others but overeating when alone, emphasizing the multidimensional nature of the contributors to guideline-discordant weight gain, including sometimes under-recognized psychological factors. By achieving a more holistic understanding of factors associated with guideline-discordant GWG, future interventions may better target modifiable psychological and behavioural mechanisms in women who are at higher risk of guideline-discordant GWG.

## Supplementary Information

**Additional file 1.** Differences in exposure variables among study participants by pregnancy weight gain status in a prospective cohort study on predictors of guideline-discordant gestational weight gain. Table of differences in exposure variables among study participants who gained inadequate, appropriate, or excess weight in a prospective cohort study on predictors of guideline-discordant gestational weight gain.

**Additional file 2.** Reliability and validity of scales used in a prospective cohort study on predictors of guideline-discordant gestational weight gain. Table of information on reliability and validity of scales used in the development of a questionnaire used in a prospective cohort study on predictors of guideline-discordant gestational weight gain.

**Additional file 3.** Univariable multinomial logistic regression analyses assessing associations between exposure variables with inadequate or excessive gestational weight gain in a prospective cohort study on predictors of guideline-discordant gestational weight gain. Table of univariable multinomial logistic regression analyses assessing associations between exposure variables with inadequate or excessive gestational weight gain in a prospective cohort study on predictors of guideline-discordant gestational weight gain.

## Data Availability

Relevant data are presented in the manuscript and its electronic supplementary material.

## Abbreviations

ANOVA: Analysis of variance
aOR: adjusted odds ratio
BMI: Body mass index
CI: Confidence interval
GWG: Gestational weight gain
IOM: Institute of Medicine
REB: Research ethics board
