# Supplementary material for: Gestational weight gain outside the 2009 Institute of Medicine recommendations: novel psychological and behavioural factors associated with inadequate or excess weight gain in a prospective cohort study
Source: BMC Pregnancy Childbirth. 2021 Jan 21;21:70. doi: 10.1186/s12884-021-03555-5 (PMC7818557; doi:10.1186/s12884-021-03555-5)
Supplement: Supplementary file 1 — Additional file 1. Differences in exposure variables among study participants by pregnancy weight gain status in a prospective cohort study on predictors of guideline-discordant gestational weight gain. Table of differences in exposure variables among study participants who gained inadequate, appropriate, or excess weight in a prospective cohort study on predictors of guideline-discordant gestational weight gain. [file 12884_2021_3555_MOESM1_ESM.docx]

| **Exposure variables** (n = 970) | | **Gestational weight gain** | | | | | | | | | | ***p*-value** |
| --- | --- | --- | --- | --- | --- | --- | --- | --- | --- | --- | --- | --- |
|  |  | **Inadequate** | | | **Appropriate** | | | | **Excess** | | |  |
|  |  | n | (n = 154) | | n | (n = 279) | | |  | (n = 537) | |  |
| **Maternal age, yr** | | 154 | 30.8 | (4.5) | 279 | | 30.9 | (5.0) | 537 | 30.2 | (4.9) | 0.097 |
| **Race** | |  |  |  |  | |  |  |  |  |  | 0.011 |
|  | White |  | 110 | (71.4) |  | | 199 | (71.3) |  | 427 | (79.5) |  |
|  | Non-white |  | 43 | (27.9) |  | | 80 | (28.7) |  | 108 | (20.1) |  |
|  | Not reported |  | 1 | (0.6) |  | | 0 | (0.0) |  | 2 | (0.4) |  |
| **Marital status** | |  |  |  |  | |  |  |  |  |  | 0.997 |
|  | Married, common-law, or living with a partner |  | 143 | (92.9) |  | | 259 | (92.8) |  | 496 | (92.4) |  |
|  | Single, divorced, or widowed |  | 11 | (7.1) |  | | 20 | (7.2) |  | 39 | (7.3) |  |
|  | Not reported |  | 0 | (0.0) |  | | 0 | (0.0) |  | 2 | (0.4) |  |
| **Education** | |  |  |  |  | |  |  |  |  |  | 0.592 |
|  | Community college or technical school or lower |  | 69 | (44.8) |  | | 112 | (40.1) |  | 231 | (43.0) |  |
|  | Undergraduate university or higher |  | 85 | (55.2) |  | | 167 | (59.9) |  | 305 | (56.8) |  |
|  | Not reported |  | 0 | (0.0) |  | | 0 | (0.0) |  | 1 | (0.2) |  |
| **Household income** | |  |  |  |  | |  |  |  |  |  | 0.450 |
|  | < $40,000 |  | 28 | (18.2) |  | | 45 | (16.1) |  | 79 | (14.7) |  |
|  | $40,000 - $79,999 |  | 39 | (25.3) |  | | 64 | (22.9) |  | 147 | (27.4) |  |
|  | ≥ $80,000 |  | 75 | (48.7) |  | | 149 | (53.4) |  | 251 | (46.7) |  |
|  | Not reported |  | 12 | (7.8) |  | | 21 | (7.5) |  | 60 | (11.2) |  |
| **Smoking** | |  |  |  |  | |  |  |  |  |  | 0.015 |
|  | Never |  | 128 | (83.1) |  | | 235 | (84.2) |  | 414 | (77.1) |  |
|  | Before this pregnancy |  | 12 | (7.8) |  | | 27 | (9.7) |  | 85 | (15.8) |  |
|  | During this pregnancy |  | 14 | (9.1) |  | | 15 | (5.4) |  | 38 | (7.1) |  |
|  | Not reported |  | 0 | (0.0) |  | | 2 | (0.7) |  | 0 | (0.0) |  |
| **Parity** | |  |  |  |  | |  |  |  |  |  | 0.115 |
|  | 0 |  | 72 | (46.8) |  | | 138 | (49.5) |  | 296 | (55.1) |  |
|  | 1+ |  | 81 | (52.6) |  | | 139 | (49.8) |  | 239 | (44.5) |  |
|  | Not reported |  | 1 | (0.6) |  | | 2 | (0.7) |  | 2 | (0.4) |  |
| **Prepregnancy BMI** | |  |  |  |  | |  |  |  |  |  | <0.001 |
|  | Underweight (BMI <18.5 kg/m^2^) |  | 6 | (3.9) |  | | 15 | (5.4) |  | 8 | (1.5) |  |
|  | Normal weight (BMI 18.5 - 24.9 kg/m^2^) |  | 101 | (65.6) |  | | 174 | (62.4) |  | 218 | (40.6) |  |
|  | Overweight (BMI 25.0 - 29.9 kg/m^2^) |  | 10 | (6.5) |  | | 46 | (16.5) |  | 176 | (32.8) |  |
|  | Obese (BMI ≥30 kg/m^2^) |  | 37 | (24.0) |  | | 44 | (15.8) |  | 135 | (25.1) |  |
| **Depression** | |  |  |  |  | |  |  |  |  |  | 0.022 |
|  | No |  | 141 | (91.6) |  | | 270 | (96.8) |  | 494 | (92.0) |  |
|  | Yes |  | 13 | (8.4) |  | | 9 | (3.2) |  | 43 | (8.0) |  |
| **Anxiety** | |  |  |  |  | |  |  |  |  |  | 0.001 |
|  | No |  | 127 | (82.5) |  | | 262 | (93.9) |  | 475 | (88.5) |  |
|  | Yes |  | 27 | (17.5) |  | | 17 | (6.1) |  | 62 | (11.5) |  |
| **Other chronic health conditions** | |  |  |  |  | |  |  |  |  |  | 0.685 |
|  | No |  | 109 | (70.8) |  | | 208 | (74.6) |  | 390 | (72.6) |  |
|  | Yes |  | 45 | (29.2) |  | | 71 | (25.4) |  | 147 | (27.4) |  |
| **Satisfied with weight before pregnancy** | |  |  |  |  | |  |  |  |  |  | 0.001 |
|  | Not very satisfied or not satisfied at all |  | 49 | (31.8) |  | | 76 | (27.2) |  | 215 | (40.0) |  |
|  | Somewhat satisfied or very satisfied |  | 104 | (67.5) |  | | 199 | (71.3) |  | 319 | (59.4) |  |
|  | Not reported |  | 1 | (0.6) |  | | 4 | (1.4) |  | 3 | (0.6) |  |
| **Planned total gestational weight gain** | |  |  |  |  | |  |  |  |  |  | <0.001 |
|  | Not reported |  | 17 | (11.0) |  | | 14 | (5.0) |  | 34 | (6.3) |  |
|  | Within guidelines |  | 43 | (27.9) |  | | 122 | (43.7) |  | 171 | (31.8) |  |
|  | Below guidelines |  | 71 | (46.1) |  | | 88 | (31.5) |  | 110 | (20.5) |  |
|  | Above guidelines |  | 23 | (14.9) |  | | 55 | (19.7) |  | 222 | (41.3) |  |
| **Weight gain recommendation levels by healthcare provider** | |  |  |  |  | |  |  |  |  |  | 0.270 |
|  | None |  | 118 | (76.6) |  | | 220 | (78.9) |  | 424 | (79.0) |  |
|  | Within guidelines |  | 16 | (10.4) |  | | 28 | (10.0) |  | 38 | (7.1) |  |
|  | Below guidelines |  | 9 | (5.8) |  | | 7 | (2.5) |  | 15 | (2.8) |  |
|  | Above guidelines |  | 3 | (1.9) |  | | 9 | (3.2) |  | 24 | (4.5) |  |
|  | Not reported/I can’t remember |  | 8 | (5.2) |  | | 15 | (5.4) |  | 36 | (6.7) |  |
| **Perceived weight gain recommendation for the 1^st^ trimester** | |  |  |  |  | |  |  |  |  |  | 0.395 |
|  | None |  | 7 | (4.5) |  | | 7 | (2.5) |  | 17 | (3.2) |  |
|  | Within guidelines |  | 50 | (32.5) |  | | 83 | (29.7) |  | 142 | (26.4) |  |
|  | Outside guidelines |  | 97 | (63.0) |  | | 189 | (67.7) |  | 378 | (70.4) |  |
| **Do you believe that there are any risks to you by gaining too little weight during pregnancy?** | |  |  |  |  | |  |  |  |  |  | 0.566 |
|  | Yes |  | 60 | (39.0) |  | | 114 | (40.9) |  | 231 | (43.0) |  |
|  | No |  | 94 | (61.0) |  | | 164 | (58.8) |  | 301 | (56.1) |  |
|  | Not reported |  | 0 | (0.0) |  | | 1 | (0.4) |  | 5 | (0.9) |  |
| **Do you believe that there are any risks to the baby by you gaining too little weight during pregnancy?** | |  |  |  |  | |  |  |  |  |  | 0.705 |
|  | Yes |  | 28 | (18.2) |  | | 56 | (20.1) |  | 113 | (21.0) |  |
|  | No |  | 126 | (81.8) |  | | 221 | (79.2) |  | 419 | (78.0) |  |
|  | Not reported |  | 0 | (0.0) |  | | 2 | (0.7) |  | 5 | (0.9) |  |
| **Do you believe that there are any risks to you by gaining too much weight during pregnancy?** | |  |  |  |  | |  |  |  |  |  | 0.841 |
|  | Yes |  | 11 | (7.1) |  | | 16 | (5.7) |  | 35 | (6.5) |  |
|  | No |  | 143 | (92.9) |  | | 261 | (93.5) |  | 497 | (92.6) |  |
|  | Not reported |  | 0 | (0.0) |  | | 2 | (0.7) |  | 5 | (0.9) |  |
| **Do you believe that there any risks to the baby by you gaining too much weight during pregnancy?** | |  |  |  |  | |  |  |  |  |  | 0.743 |
|  | Yes |  | 14 | (9.1) |  | | 32 | (11.5) |  | 57 | (10.6) |  |
|  | No |  | 139 | (90.3) |  | | 245 | (87.8) |  | 475 | (88.5) |  |
|  | Not reported |  | 1 | (0.6) |  | | 2 | (0.7) |  | 5 | (0.9) |  |
| **Whether my weight changes is up to me** | |  |  |  |  | |  |  |  |  |  | 0.820 |
|  | Disagree or strongly disagree |  | 50 | (32.5) |  | | 88 | (31.5) |  | 190 | (35.4) |  |
|  | Neither disagree nor agree |  | 44 | (28.6) |  | | 74 | (26.5) |  | 138 | (25.7) |  |
|  | Agree or strongly agree |  | 59 | (38.3) |  | | 113 | (40.5) |  | 205 | (38.2) |  |
|  | Not reported |  | 1 | (0.6) |  | | 4 | (1.4) |  | 4 | (0.7) |  |
| **If I eat right, and can get enough exercise and rest, I can control my weight the way I want** | |  |  |  |  | |  |  |  |  |  | 0.105 |
|  | Disagree or strongly disagree |  | 34 | (22.1) |  | | 37 | (13.3) |  | 99 | (18.4) |  |
|  | Neither disagree nor agree |  | 30 | (19.5) |  | | 48 | (17.2) |  | 106 | (19.7) |  |
|  | Agree or strongly agree |  | 90 | (58.4) |  | | 191 | (68.5) |  | 330 | (61.5) |  |
|  | Not reported |  | 0 | (0.0) |  | | 3 | (1.1) |  | 2 | (0.4) |  |
| **Being the right weight is mainly good luck** | |  |  |  |  | |  |  |  |  |  | 0.998 |
|  | Agree or strongly agree |  | 12 | (7.8) |  | | 22 | (7.9) |  | 41 | (7.6) |  |
|  | Neither disagree nor agree |  | 29 | (18.8) |  | | 52 | (18.6) |  | 106 | (19.7) |  |
|  | Disagree or strongly disagree |  | 110 | (71.4) |  | | 201 | (72.0) |  | 386 | (71.9) |  |
|  | Not reported |  | 3 | (1.9) |  | | 4 | (1.4) |  | 4 | (0.7) |  |
| **You can’t control the amount of weight you gain when you are pregnant** | |  |  |  |  | |  |  |  |  |  | 0.310 |
|  | Agree or strongly agree |  | 19 | (12.3) |  | | 32 | (11.5) |  | 86 | (16.0) |  |
|  | Neither disagree nor agree |  | 30 | (19.5) |  | | 57 | (20.4) |  | 118 | (22.0) |  |
|  | Disagree or strongly disagree |  | 104 | (67.5) |  | | 187 | (67.0) |  | 330 | (61.5) |  |
|  | Not reported |  | 1 | (0.6) |  | | 3 | (1.1) |  | 3 | (0.6) |  |
| **Think that family and friends believe that pregnant women need to eat two times as much as before pregnancy** | |  |  |  |  | |  |  |  |  |  | 0.244 |
|  | Disagree or strongly disagree |  | 117 | (76.0) |  | | 210 | (75.3) |  | 417 | (77.7) |  |
|  | Neither disagree nor agree |  | 16 | (10.4) |  | | 35 | (12.5) |  | 43 | (8.0) |  |
|  | Agree or strongly agree |  | 21 | (13.6) |  | | 31 | (11.1) |  | 76 | (14.2) |  |
|  | Not reported |  | 0 | (0.0) |  | | 3 | (1.1) |  | 1 | (0.2) |  |
| **Think that family and friends believe that pregnant women crave foods more intensely than other people** | |  |  |  |  | |  |  |  |  |  | 0.180 |
|  | Disagree or strongly disagree |  | 26 | (16.9) |  | | 30 | (10.8) |  | 55 | (10.2) |  |
|  | Neither disagree nor agree |  | 21 | (13.6) |  | | 44 | (15.8) |  | 96 | (17.9) |  |
|  | Agree or strongly agree |  | 107 | (69.5) |  | | 201 | (72.0) |  | 383 | (71.3) |  |
|  | Not reported |  | 0 | (0.0) |  | | 4 | (1.4) |  | 3 | (0.6) |  |
| **Think that family and friends believe that pregnant women should eat what they crave** | |  |  |  |  | |  |  |  |  |  | 0.926 |
|  | Disagree or strongly disagree |  | 50 | (32.5) |  | | 86 | (30.8) |  | 177 | (33.0) |  |
|  | Neither disagree nor agree |  | 61 | (39.6) |  | | 102 | (36.6) |  | 197 | (36.7) |  |
|  | Agree or strongly agree |  | 43 | (27.9) |  | | 87 | (31.2) |  | 162 | (30.2) |  |
|  | Not reported |  | 0 | (0.0) |  | | 4 | (1.4) |  | 1 | (0.2) |  |
| **Think that family and friends believe that pregnant women should not exert themselves physically** | |  |  |  |  | |  |  |  |  |  | 0.056 |
|  | Disagree or strongly disagree |  | 60 | (39.0) |  | | 86 | (30.8) |  | 203 | (37.8) |  |
|  | Neither disagree nor agree |  | 21 | (13.6) |  | | 51 | (18.3) |  | 110 | (20.5) |  |
|  | Agree or strongly agree |  | 72 | (46.8) |  | | 138 | (49.5) |  | 220 | (41.0) |  |
|  | Not reported |  | 1 | (0.6) |  | | 4 | (1.4) |  | 4 | (0.7) |  |
| **Think that family and friends believe that pregnant women should not be worried about gaining too much weight during pregnancy** | |  |  |  |  | |  |  |  |  |  | 0.328 |
|  | Disagree or strongly disagree |  | 75 | (48.7) |  | | 146 | (52.3) |  | 265 | (49.3) |  |
|  | Neither disagree nor agree |  | 26 | (16.9) |  | | 57 | (20.4) |  | 95 | (17.7) |  |
|  | Agree or strongly agree |  | 53 | (34.4) |  | | 72 | (25.8) |  | 173 | (32.2) |  |
|  | Not reported |  | 0 | (0.0) |  | | 4 | (1.4) |  | 4 | (0.7) |  |
| **How often do you eat meals in front of a screen?** | |  |  |  |  | |  |  |  |  |  | <0.001 |
|  | None or almost no meals |  | 53 | (34.4) |  | | 114 | (40.9) |  | 141 | (26.3) |  |
|  | Some meals |  | 68 | (44.2) |  | | 120 | (43.0) |  | 295 | (54.9) |  |
|  | Most meals or more |  | 32 | (20.8) |  | | 40 | (14.3) |  | 93 | (17.3) |  |
|  | Not reported |  | 1 | (0.6) |  | | 5 | (1.8) |  | 8 | (1.5) |  |
| **How often do you watch television before going to sleep?** | |  |  |  |  | |  |  |  |  |  | 0.577 |
|  | None |  | 94 | (61.0) |  | | 180 | (64.5) |  | 318 | (59.2) |  |
|  | Some nights |  | 26 | (16.9) |  | | 45 | (16.1) |  | 105 | (19.6) |  |
|  | Most nights or more |  | 34 | (22.1) |  | | 51 | (18.3) |  | 106 | (19.7) |  |
|  | Not reported |  | 0 | (0.0) |  | | 3 | (1.1) |  | 8 | (1.5) |  |
| **During a typical day, do you drink soda pop, cola, or juice?** | |  |  |  |  | |  |  |  |  |  | 0.744 |
|  | No |  | 92 | (59.7) |  | | 162 | (58.1) |  | 303 | (56.4) |  |
|  | Yes |  | 62 | (40.3) |  | | 116 | (41.6) |  | 233 | (43.4) |  |
|  | Not reported |  | 0 | (0.0) |  | | 1 | (0.4) |  | 1 | (0.2) |  |
| **On average, how many times would you eat fast food?** | |  |  |  |  | |  |  |  |  |  | 0.056 |
|  | ≤1 time/month |  | 61 | (39.6) |  | | 93 | (33.3) |  | 149 | (27.7) |  |
|  | 2-3 times/month |  | 46 | (29.9) |  | | 99 | (35.5) |  | 202 | (37.6) |  |
|  | ≥1 time/week |  | 47 | (30.5) |  | | 85 | (30.5) |  | 186 | (34.6) |  |
|  | Not reported |  | 0 | (0.0) |  | | 2 | (0.7) |  | 0 | (0.0) |  |
| **Fruit and vegetable intake per day** | |  |  |  |  | |  |  |  |  |  | 0.835 |
|  | < 5 servings/day |  | 87 | (56.5) |  | | 151 | (54.1) |  | 291 | (54.2) |  |
|  | ≥ 5 servings/day |  | 64 | (41.6) |  | | 123 | (44.1) |  | 239 | (44.5) |  |
|  | Not reported |  | 3 | (1.9) |  | | 5 | (1.8) |  | 7 | (1.3) |  |
| **On average, how many times would you eat snack foods?** | |  |  |  |  | |  |  |  |  |  | 0.563 |
|  | ≤1 time/week |  | 68 | (44.2) |  | | 125 | (44.8) |  | 223 | (41.5) |  |
|  | ≥2 times/week |  | 86 | (55.8) |  | | 151 | (54.1) |  | 314 | (58.5) |  |
|  | Not reported |  | 0 | (0.0) |  | | 3 | (1.1) |  | 0 | (0.0) |  |
| **How much of your daily food intake do you eat after suppertime?** | |  |  |  |  | |  |  |  |  |  | 0.947 |
|  | <¼ |  | 143 | (92.9) |  | | 256 | (91.8) |  | 501 | (93.3) |  |
|  | ≥¼ |  | 10 | (6.5) |  | | 20 | (7.2) |  | 36 | (6.7) |  |
|  | Not reported |  | 1 | (0.6) |  | | 3 | (1.1) |  | 0 | (0.0) |  |
| **Do you snack in the middle of the night?** | |  |  |  |  | |  |  |  |  |  | 0.334 |
|  | No |  | 128 | (83.1) |  | | 249 | (89.2) |  | 465 | (86.6) |  |
|  | Yes |  | 23 | (14.9) |  | | 29 | (10.4) |  | 69 | (12.8) |  |
|  | Not reported |  | 3 | (1.9) |  | | 1 | (0.4) |  | 3 | (0.6) |  |
| **During this pregnancy, do you have feelings of guilt after overeating?** | |  |  |  |  | |  |  |  |  |  | 0.571 |
|  | Never or rarely |  | 123 | (79.9) |  | | 224 | (80.3) |  | 430 | (80.1) |  |
|  | Often or always |  | 23 | (14.9) |  | | 42 | (15.1) |  | 97 | (18.1) |  |
|  | Not reported |  | 8 | (5.2) |  | | 13 | (4.7) |  | 10 | (1.9) |  |
| **During this pregnancy, do you ever feel that when you started eating you just couldn’t stop?** | |  |  |  |  | |  |  |  |  |  | 0.259 |
|  | Never or rarely |  | 134 | (87.0) |  | | 249 | (89.2) |  | 470 | (87.5) |  |
|  | Often or always |  | 13 | (8.4) |  | | 19 | (6.8) |  | 56 | (10.4) |  |
|  | Not reported |  | 7 | (4.5) |  | | 11 | (3.9) |  | 11 | (2.0) |  |
| **During pregnancy, you can eat foods that are good for you, even when family or social life takes a lot of your time** | |  |  |  |  | |  |  |  |  |  | 0.536 |
|  | Unsure or very unsure |  | 6 | (3.9) |  | | 16 | (5.7) |  | 38 | (7.1) |  |
|  | Neither unsure or sure |  | 13 | (8.4) |  | | 29 | (10.4) |  | 58 | (10.8) |  |
|  | Sure or very sure |  | 134 | (87.0) |  | | 232 | (83.2) |  | 439 | (81.8) |  |
|  | Not reported |  | 1 | (0.6) |  | | 2 | (0.7) |  | 2 | (0.4) |  |
| **During pregnancy, you can get regular exercise** | |  |  |  |  | |  |  |  |  |  | 0.185 |
|  | Unsure or very unsure |  | 24 | (15.6) |  | | 28 | (10.0) |  | 63 | (11.7) |  |
|  | Neither unsure or sure |  | 13 | (8.4) |  | | 37 | (13.3) |  | 78 | (14.5) |  |
|  | Sure or very sure |  | 115 | (74.7) |  | | 213 | (76.3) |  | 394 | (73.4) |  |
|  | Not reported |  | 2 | (1.3) |  | | 1 | (0.4) |  | 2 | (0.4) |  |
| **I control my emotions by not expressing them** | |  |  |  |  | |  |  |  |  |  | 0.222 |
|  | Almost never or sometimes |  | 108 | (70.1) |  | | 217 | (77.8) |  | 415 | (77.3) |  |
|  | About half the time |  | 34 | (22.1) |  | | 40 | (14.3) |  | 78 | (14.5) |  |
|  | Most of the time or almost always |  | 12 | (7.8) |  | | 22 | (7.9) |  | 42 | (7.8) |  |
|  | Not reported |  | 0 | (0.0) |  | | 0 | (0.0) |  | 2 | (0.4) |  |
| **When I am upset, I have difficulty controlling my behaviour** | |  |  |  |  | |  |  |  |  |  | 0.995 |
|  | Almost never or sometimes |  | 127 | (82.5) |  | | 234 | (83.9) |  | 450 | (83.8) |  |
|  | About half the time |  | 15 | (9.7) |  | | 25 | (9.0) |  | 47 | (8.8) |  |
|  | Most of the time or almost always |  | 9 | (5.8) |  | | 18 | (6.5) |  | 33 | (6.1) |  |
|  | Not reported |  | 3 | (1.9) |  | | 2 | (0.7) |  | 7 | (1.3) |  |
| **When I’m upset, it takes me a long time to feel better** | |  |  |  |  | |  |  |  |  |  | 0.457 |
|  | Almost never or sometimes |  | 117 | (76.0) |  | | 223 | (79.9) |  | 421 | (78.4) |  |
|  | About half the time |  | 24 | (15.6) |  | | 34 | (12.2) |  | 65 | (12.1) |  |
|  | Most of the time or almost always |  | 11 | (7.1) |  | | 16 | (5.7) |  | 47 | (8.8) |  |
|  | Not reported |  | 2 | (1.3) |  | | 6 | (2.2) |  | 4 | (0.7) |  |
| **When I’m upset, I believe that there’s nothing I can do to make myself feel better** | |  |  |  |  | |  |  |  |  |  | 0.433 |
|  | Almost never or sometimes |  | 145 | (94.2) |  | | 254 | (91.0) |  | 497 | (92.6) |  |
|  | About half the time |  | 5 | (3.2) |  | | 20 | (7.2) |  | 30 | (5.6) |  |
|  | Most of the time or almost always |  | 4 | (2.6) |  | | 4 | (1.4) |  | 8 | (1.5) |  |
|  | Not reported |  | 0 | (0.0) |  | | 1 | (0.4) |  | 2 | (0.4) |  |
| **When I'm upset, I know I can find a way to eventually feel better** | |  |  |  |  | |  |  |  |  |  | 0.242 |
|  | Most of the time or almost always |  | 120 | (77.9) |  | | 237 | (84.9) |  | 431 | (80.3) |  |
|  | About half the time |  | 13 | (8.4) |  | | 22 | (7.9) |  | 51 | (9.5) |  |
|  | Almost never or sometimes |  | 20 | (13.0) |  | | 19 | (6.8) |  | 52 | (9.7) |  |
|  | Not reported |  | 1 | (0.6) |  | | 1 | (0.4) |  | 3 | (0.6) |  |
| **When I am upset, I become embarrassed for feeling that way** | |  |  |  |  | |  |  |  |  |  | 0.685 |
|  | Almost never or sometimes |  | 122 | (79.2) |  | | 230 | (82.4) |  | 433 | (80.6) |  |
|  | About half the time |  | 15 | (9.7) |  | | 30 | (10.8) |  | 59 | (11.0) |  |
|  | Most of the time or almost always |  | 16 | (10.4) |  | | 18 | (6.5) |  | 42 | (7.8) |  |
|  | Not reported |  | 1 | (0.6) |  | | 1 | (0.4) |  | 3 | (0.6) |  |
| **I feel that I must do things perfectly or not do them at all** | |  |  |  |  | |  |  |  |  |  | 0.139 |
|  | Almost never or sometimes |  | 120 | (77.9) |  | | 206 | (73.8) |  | 389 | (72.4) |  |
|  | About half the time |  | 10 | (6.5) |  | | 40 | (14.3) |  | 74 | (13.8) |  |
|  | Most of the time or almost always |  | 22 | (14.3) |  | | 32 | (11.5) |  | 71 | (13.2) |  |
|  | Not reported |  | 2 | (1.3) |  | | 1 | (0.4) |  | 3 | (0.6) |  |
| **During the 3 months before pregnancy, how often were you dieting?** | |  |  |  |  | |  |  |  |  |  | 0.007 |
|  | Never or rarely |  | 131 | (85.1) |  | | 236 | (84.6) |  | 410 | (76.4) |  |
|  | Often or always |  | 23 | (14.9) |  | | 43 | (15.4) |  | 125 | (23.3) |  |
|  | Not reported |  | 0 | (0.0) |  | | 0 | (0.0) |  | 2 | (0.4) |  |
| **During the 3 months before pregnancy, how often did you limit your carbohydrate and sugar intake?** | |  |  |  |  | |  |  |  |  |  | 0.032 |
|  | Never or rarely |  | 103 | (66.9) |  | | 168 | (60.2) |  | 297 | (55.3) |  |
|  | Often or always |  | 51 | (33.1) |  | | 111 | (39.8) |  | 239 | (44.5) |  |
|  | Not reported |  | 0 | (0.0) |  | | 0 | (0.0) |  | 1 | (0.2) |  |
| **During the 3 months before pregnancy, did you have feelings of guilt after overeating?** | |  |  |  |  | |  |  |  |  |  | 0.026 |
|  | Never or rarely |  | 109 | (70.8) |  | | 203 | (72.8) |  | 343 | (63.9) |  |
|  | Often or always |  | 45 | (29.2) |  | | 76 | (27.2) |  | 193 | (35.9) |  |
|  | Not reported |  | 0 | (0.0) |  | | 0 | (0.0) |  | 1 | (0.2) |  |
| **During the 3 months before pregnancy, did you ever feel that when you started eating you just couldn’t stop?** | |  |  |  |  | |  |  |  |  |  | 0.043 |
|  | Never or rarely |  | 134 | (87.0) |  | | 259 | (92.8) |  | 471 | (87.7) |  |
|  | Often or always |  | 20 | (13.0) |  | | 19 | (6.8) |  | 65 | (12.1) |  |
|  | Not reported |  | 0 | (0.0) |  | | 1 | (0.4) |  | 1 | (0.2) |  |
| **During the 3 months before pregnancy, did you want to eat when you were emotionally upset?** | |  |  |  |  | |  |  |  |  |  | 0.002 |
|  | Never or rarely |  | 121 | (78.6) |  | | 212 | (76.0) |  | 358 | (66.7) |  |
|  | Often or always |  | 33 | (21.4) |  | | 66 | (23.7) |  | 178 | (33.1) |  |
|  | Not reported |  | 0 | (0.0) |  | | 1 | (0.4) |  | 1 | (0.2) |  |
| **I eat sensibly when with others, but overeat when I’m alone** | |  |  |  |  | |  |  |  |  |  | <0.001 |
|  | No |  | 85 | (55.2) |  | | 183 | (65.6) |  | 270 | (50.3) |  |
|  | Yes |  | 68 | (44.2) |  | | 95 | (34.1) |  | 264 | (49.2) |  |
|  | Not reported |  | 1 | (0.6) |  | | 1 | (0.4) |  | 3 | (0.6) |  |
| **If others saw how much I ate, then I’d feel ashamed** | |  |  |  |  | |  |  |  |  |  | 0.005 |
|  | No |  | 121 | (78.6) |  | | 234 | (83.9) |  | 398 | (74.1) |  |
|  | Yes |  | 32 | (20.8) |  | | 43 | (15.4) |  | 136 | (25.3) |  |
|  | Not reported |  | 1 | (0.6) |  | | 2 | (0.7) |  | 3 | (0.6) |  |
| **When I am considering eating more than I should, or when I am considering eating a food that I feel is not particularly healthy, I tell myself it is okay because I will eat healthier later** | |  |  |  |  | |  |  |  |  |  | 0.020 |
|  | Never |  | 46 | (29.9) |  | | 67 | (24.0) |  | 105 | (19.6) |  |
|  | Rarely, frequently, or always |  | 106 | (68.8) |  | | 211 | (75.6) |  | 426 | (79.3) |  |
|  | Not reported |  | 2 | (1.3) |  | | 1 | (0.4) |  | 6 | (1.1) |  |
| **When I am considering eating more than I should, or when I am considering eating a food that I feel is not particularly healthy, I tell myself it is okay because I will compensate by eating less later** | |  |  |  |  | |  |  |  |  |  | 0.754 |
|  | Never |  | 66 | (42.9) |  | | 117 | (41.9) |  | 214 | (39.9) |  |
|  | Rarely, frequently, or always |  | 86 | (55.8) |  | | 160 | (57.3) |  | 316 | (58.8) |  |
|  | Not reported |  | 2 | (1.3) |  | | 2 | (0.7) |  | 7 | (1.3) |  |
| **When I am considering eating more than I should, or when I am considering eating a food that I feel is not particularly healthy, I tell myself it is okay because I will compensate by doing some exercise later** | |  |  |  |  | |  |  |  |  |  | 0.106 |
|  | Never |  | 44 | (28.6) |  | | 79 | (28.3) |  | 121 | (22.5) |  |
|  | Rarely, frequently, or always |  | 108 | (70.1) |  | | 198 | (71.0) |  | 412 | (76.7) |  |
|  | Not reported |  | 2 | (1.3) |  | | 2 | (0.7) |  | 4 | (0.7) |  |
| **I have a lot of fear regarding the health of my baby** | |  |  |  |  | |  |  |  |  |  | 0.078 |
|  | No |  | 124 | (80.5) |  | | 204 | (73.1) |  | 386 | (71.9) |  |
|  | Yes |  | 27 | (17.5) |  | | 67 | (24.0) |  | 142 | (26.4) |  |
|  | Not reported |  | 3 | (1.9) |  | | 8 | (2.9) |  | 9 | (1.7) |  |
| **Nausea related to pregnancy** | |  |  |  |  | |  |  |  |  |  | 0.833 |
|  | Never or 1 time/week |  | 42 | (27.3) |  | | 70 | (25.1) |  | 133 | (24.8) |  |
|  | ≥ 1 time/day |  | 112 | (72.7) |  | | 209 | (74.9) |  | 401 | (74.7) |  |
|  | Not reported |  | 0 | (0.0) |  | | 0 | (0.0) |  | 3 | (0.6) |  |
| **Food cravings related to pregnancy** | |  |  |  |  | |  |  |  |  |  | 0.053 |
|  | Never or 1 time/week |  | 106 | (68.8) |  | | 170 | (60.9) |  | 338 | (62.9) |  |
|  | ≥ 1 time/day |  | 39 | (25.3) |  | | 106 | (38.0) |  | 193 | (35.9) |  |
|  | Not reported |  | 9 | (5.8) |  | | 3 | (1.1) |  | 6 | (1.1) |  |
| **Eat something to cope with nausea** | |  |  |  |  | |  |  |  |  |  | 0.608 |
|  | No |  | 73 | (47.4) |  | | 123 | (44.1) |  | 256 | (47.7) |  |
|  | Yes |  | 81 | (52.6) |  | | 156 | (55.9) |  | 281 | (52.3) |  |
| **Avoiding eating to cope with nausea** | |  |  |  |  | |  |  |  |  |  | 0.359 |
|  | No |  | 120 | (77.9) |  | | 218 | (78.1) |  | 439 | (81.8) |  |
|  | Yes |  | 34 | (22.1) |  | | 61 | (21.9) |  | 98 | (18.2) |  |
| **Take medication to cope with nausea** | |  |  |  |  | |  |  |  |  |  | 0.160 |
|  | None |  | 33 | (21.4) |  | | 48 | (17.2) |  | 114 | (21.2) |  |
|  | Medication use |  | 7 | (4.5) |  | | 26 | (9.3) |  | 54 | (10.1) |  |
|  | Other ways |  | 114 | (74.0) |  | | 205 | (73.5) |  | 369 | (68.7) |  |
| **Eat what I crave to cope with cravings** | |  |  |  |  | |  |  |  |  |  | 0.657 |
|  | No |  | 51 | (33.1) |  | | 81 | (29.0) |  | 160 | (29.8) |  |
|  | Yes |  | 103 | (66.9) |  | | 198 | (71.0) |  | 377 | (70.2) |  |
| **Avoid what I crave to cope with cravings** | |  |  |  |  | |  |  |  |  |  | 0.980 |
|  | No |  | 135 | (87.7) |  | | 243 | (87.1) |  | 470 | (87.5) |  |
|  | Yes |  | 19 | (12.3) |  | | 36 | (12.9) |  | 67 | (12.5) |  |
| **Distract myself to cope with cravings** | |  |  |  |  | |  |  |  |  |  | 0.774 |
|  | No |  | 127 | (82.5) |  | | 225 | (80.6) |  | 429 | (79.9) |  |
|  | Yes |  | 27 | (17.5) |  | | 54 | (19.4) |  | 108 | (20.1) |  |
| **Sleep** | |  |  |  |  | |  |  |  |  |  | 0.625 |
|  | < 8 hr/day |  | 63 | (40.9) |  | | 126 | (45.2) |  | 222 | (41.3) |  |
|  | ≥ 8 hr/day |  | 86 | (55.8) |  | | 153 | (54.8) |  | 311 | (57.9) |  |
|  | Not reported |  | 5 | (3.2) |  | | 0 | (0.0) |  | 4 | (0.7) |  |
| **Total physical activity** | |  |  |  |  | |  |  |  |  |  | 0.342 |
|  | Low |  | 55 | (35.7) |  | | 84 | (30.1) |  | 158 | (29.4) |  |
|  | Medium |  | 37 | (24.0) |  | | 89 | (31.9) |  | 173 | (32.2) |  |
|  | High |  | 47 | (30.5) |  | | 90 | (32.3) |  | 161 | (30.0) |  |
|  | Not reported |  | 15 | (9.7) |  | | 16 | (5.7) |  | 45 | (8.4) |  |
| **Preferred body size image before pregnancy** | |  |  |  |  | |  |  |  |  |  | 0.012 |
|  | Underweight |  | 40 | (26.0) |  | | 53 | (19.0) |  | 75 | (14.0) |  |
|  | Normal weight |  | 104 | (67.5) |  | | 203 | (72.8) |  | 421 | (78.4) |  |
|  | Overweight or obese |  | 10 | (6.5) |  | | 21 | (7.5) |  | 38 | (7.1) |  |
|  | Not reported |  | 0 | (0.0) |  | | 2 | (0.7) |  | 3 | (0.6) |  |
| **Comparison between perceived and preferred body size image** | |  |  |  |  | |  |  |  |  |  | 0.013 |
|  | Actually smaller than preferred |  | 6 | (3.9) |  | | 20 | (7.2) |  | 20 | (3.7) |  |
|  | Accurate |  | 54 | (35.1) |  | | 97 | (34.8) |  | 148 | (27.6) |  |
|  | Actually larger than preferred |  | 94 | (61.0) |  | | 160 | (57.3) |  | 366 | (68.2) |  |
|  | Not reported |  | 0 | (0.0) |  | | 2 | (0.7) |  | 3 | (0.6) |  |
| **Comparison between BMI and perceived body size image** | |  |  |  |  | |  |  |  |  |  | <0.001 |
|  | Actually smaller than perceived |  | 6 | (3.9) |  | | 13 | (4.7) |  | 13 | (2.4) |  |
|  | Accurate |  | 94 | (61.0) |  | | 179 | (64.2) |  | 270 | (50.3) | a |
|  | Actually larger than perceived |  | 54 | (35.1) |  | | 86 | (30.8) |  | 251 | (46.7) |  |
|  | Not reported |  | 0 | (0.0) |  | | 1 | (0.4) |  | 3 | (0.6) |  |
| **Sitting time/day, hr** | | 139 | 6.4 | (3.2) | 279 | | 30.9 | (5.0) | 537 | 30.2 | (4.9) | 0.742 |
| **TPB score** | | 152 | 3.8 | (0.9) | 263 | | 6.5 | (3.1) | 492 | 6.6 | (3.1) | 0.940 |
| **Personality- Extraversion** | | 149 | 4.4 | (1.4) | 278 | | 3.9 | (1.0) | 533 | 3.8 | (1.0) | 0.690 |
| **Personality- Agreeableness** | | 147 | 5.1 | (1.0) | 274 | | 4.3 | (1.5) | 529 | 4.4 | (1.5) | 0.024 |
| **Personality- Conscientiousness** | | 148 | 5.8 | (1.0) | 272 | | 5.3 | (1.0) | 526 | 5.4 | (1.0) | 0.064 |
| **Personality- Emotional Stability** | | 148 | 4.7 | (1.4) | 273 | | 5.8 | (1.0) | 530 | 5.6 | (1.1) | 0.467 |
| **Personality- Openness** | | 149 | 5.1 | (1.1) | 275 | | 4.9 | (1.3) | 530 | 4.8 | (1.3) | 0.987 |
| Data are means (standard deviation) and number of participants (percentage). Percentages may not total 100 due to rounding. The descriptive and univariable multinomial logistic regression analyses were complete case analyses.  BMI, body mass index; TPB, theory of planned behavior. | | | | | | | | | | | | |
