# Supplementary material for: Gestational weight gain outside the 2009 Institute of Medicine recommendations: novel psychological and behavioural factors associated with inadequate or excess weight gain in a prospective cohort study
Source: BMC Pregnancy Childbirth. 2021 Jan 21;21:70. doi: 10.1186/s12884-021-03555-5 (PMC7818557; doi:10.1186/s12884-021-03555-5)
Supplement: Supplementary file 2 — Additional file 2. Reliability and validity of scales used in a prospective cohort study on predictors of guideline-discordant gestational weight gain. Table of information on reliability and validity of scales used in the development of a questionnaire used in a prospective cohort study on predictors of guideline-discordant gestational weight gain. [file 12884_2021_3555_MOESM2_ESM.docx]

| **Type of factor** | **Focus of the scale focus, reference** | **Reliability: Cronbach’s alpha and test-retest** | **Validity** |
| --- | --- | --- | --- |
| **Cognition** | **The Dutch Eating Behavior Questionnaire**  **Emotional eating item**  Van Strien et al.^1^ | Emotional Eating item is part of 13-item Emotional eating scale, with an **Item-total correlation:** α = 0.71 | "The norms and Cronbach's alpha coefficients of the scales and also the Pearson's correlation coefficients to assess interrelationships between scales indicate that the scales have a**high internal consistency and factorial validity**." |
| **Cognition** | **Revised dietary restraint scale**  **Modified concern with dieting subscale**  Originally by Herman et al.^2^, Modified by van Strien et al.^3^ | **Cronbach's α** = 0.77. | **Construct validity:** high factor loadings indicated CD indeed measures restrained eating |
| **Cognition** | **Disinhibitive Eating Scale**  **Bingeing subscale**  Overduin & Jansen^4^ | **Cronbach's α** = 0.88 | **Content validity**: suggested by research of Tobin, Johnson, Steinberg, Staats and Dennis (1991) who factor analyzed a large and complete self-report battery on bulimic symptoms in patients: four factors of the six factors found, i.e. weight fluctuations, binge eating, self-esteem and failed restriction are also present within DIS. |
| **Cognition** | **Normative beliefs on pregnancy weight gain, diet, and exercise;** No existing scales for pregnancy found in our systematic review therefore we developed the questions based on postpartum scales (Hales et al ^5^) | Investigator developed | **Content validity:** based on psychologists’ and obstetrician’s previously published work (Hales et al^5^) |
| **Cognition** | **Barriers to healthy eating scale** (BHES)  **Expense of Healthy Eating subscale**  Fowles & Feucht^6^ | **Cronbach’s α** not mentioned;  **Test-retest reliability**: for expense subscale correlation 0.91( *p* < .01) at time 1 and two weeks later at time 2 | **Content validity**: panel of nutrition experts assessed the relevance of the items to conceptual elements of the scale and subscales using rankings, and the resultant “content validity index of the 16-item scale was .75  **Criterion validity**: Assessed by correlating total and subscale scores of the BHES with scores on the Nutrition subscale of the Health Promoting Lifestyle Profile-II (HPLP-2), as anticipated, a negative relationship existed; for expense subscale correlation -.07, 0.74 (p<.01)  **Construct validity**  Factor analysis resulted in a 5-factor scale that explained 73% of the variance.  Kaiser’s criterion of using all unrotated factors with eigenvalues greater than 1.0 was applied. |
| **Cognition** | **Self-efficacy**  **Control of food intake and exercise subscales**  Kendall et al.^7^ | **Cronbach’s α** = 0.80 for control of food intake, 0.94 for exercise | **Content validity:** as basis for scale development, used Hofstetter et al 1990 (Some health dimensions of self-efficacy) |
| **Cognition** | **Weight locus of control** (adapted from Saltzer^8^) and **Weight attitudes** (informed by Palmer^9^)  Kendall et al.^7^ | **Cronbach’s α** for weight locus of control (0.71 Internality, 0.69 Externality) ^7^; weight attitudes (negative 0.80; healthy 0.65) ^7^ | **Content validity:** assessed using factor analysis  **Construct validity:** using hypothesis testing  --locus of control compared using t test (Mean for successful weight loss 4.13, SEM 0.59, p 0.0001)  --weight attitudes: scores were used as the dependant variables in the general linear models procedure in SAS (β coefficient with BMI -0.03, SE 0.004, p=0.0001) |
| **Cognition** | **Self-Motivation Inventory**  **Goal striving subscale**  Dishman & Ickes^10^ | **Cronbach’s α** = 0.78 for goal striving subscale  **Test-retest correlation coefficient for overall 40-item Self-Motivation Inventory**:*r*=0.92 over 1-month | Scale has consistently positive predicts subsequent **success in weight control**^11^ ,^12^. |
| **Cognition** | **Nutrition knowledge**  **Choosing foods subscale**  Parmenter & Wardle^13^ | **Cronbach’s α** = 0.76 for choosing foods  **Test-retest reliability**: Pearson’s correlation for 105 respondents who completed twice: 0.87 for choosing foods | **Construct** **validity**: dietetic students scored higher than computer science students (p<0.001)  81.4 for choosing foods; met construct validity overall |
| **Cognition** | **Body Image**  **CBS** (**current body size**) and **IBS** (**ideal body size**), CBS minus IBS (CBS-IBS) is a measure of **body size dissatisfaction**  Williamson et al.^14^ | **Cronbach’s α** not mentioned;  **Test-retest** (two weeks apart) Pearson correlation coefficient, r = 0.93 for CBS, r =0.77 for IBS (N=77 ) | **Criterion validity:** discrepancy between CBS, IBS estimates was supported by positive correlations with two measures of body dissatisfaction. Compared to Body Shape Questionnaire (BSQ) by Cooper et al. (intercorrelation with CBS - IBS and BSQ is 0.454, p<0.01) and the Body Dissatisfaction scale as part of the Eating Disorder Inventory-2 (intercorrelation with CBS - IBS and BSQ is 0.475, p<0.01) |
| **Cognition** | **Personal Target, and Actual Weight**  Cogswell et al.^15^ | **Cronbach’s α** not mentioned | Validation not mentioned |
| **Affect** | **Patient Health Questionnaire**  Brief Screen Scale for **Anxiety and Depression**: PHQ-4  Kroenke et al.^16^ | **Cronbach’s α** = 0.85 | A cut point of ≥3 on the 0-to-6-point PHQ-2 scale has a sensitivity of 83% and specificity of 90% for major depressive disorder.^17^ (A cutpoint of ≥3 on the 0-to-6-point GAD-2has 88% sensitivity for generalized anxiety disorder, 76% for panic disorder, and 70% for social anxiety disorder.^18^ |
| **Affect** | **Edinburgh Postnatal Depression Scale**  **Self-harm item**  Cox et al.^19^ | **Cronbach’s α** =0.87 | A cut-off score of >10 resulted in: sensitivity of 85%, specificity of 77%, positive predictive value of 83% |
| **Affect** | **Pregnancy-Related Anxiety**  Rini et al.^20^ | **Cronbach's α** = .78 | Conducted exploratory factor analysis with oblique rotation to investigate the factor structure of these items. Examination of the eigenvalues revealed that the scores were best represented by a single factor. |
| **Personality** | **Ten Item Personality Inventory (assessing five factor model)**  Gosling^21^ | **Since there are only 2 items per subscale, rather than emphasizing Cronbach α they emphasized content validity. Cronbach's α** (Extroversion) = 0.68, Agreeableness = 0.40, Conscientiousness = 0.50, Emotional Instability = 0.73, Openness to Experience = 0.45  **Test-retest correlation**, mean *r* = 0.72. | The TIPI displayed patterns of correlations that were “virtually identical to those of the (44 item) Big Five Inventory (Benet-Martinez & John 1998); with all column-vector correlations exceeding 0.90."  **Convergences** that were comparable to other multi-item scales (mean r=0.77) |
| **Personality** | **Emotion Regulation Questionnaire**  **Suppression subscale**  Gross & John^22^ | **Cronbach's α** =0.73 for suppression | **Construct validity**: convergent and discriminant; only *indirect evidence* for the construct validity of the suppression scale.  **--**correlated with the NMR |
| **Personality** | **Difficulties in Emotion Regulation** Scale (DERS)  **Impulse control and difficulties;**  **Nonacceptance;**  **Strategies** **subscales**  Gratz & Roemer^23^ | **Cronbach's α =**0.93 for total scale  **Cronbach's α** =0.86 for impulse control subscale , 0.85 for nonacceptance, 0.88 for strategies  **Test-retest** reliability over a period ranging from 4-8 weeks = .88, *p* <.01, = .57 for **impulse control** | **Construct validity:** compared to NMR, a commonly used measure of emotion regulation; overall DERS in the expected directed and *statistically significant* (.69**; **=p<.01) as were each of the DERS subscales (nonacceptance .42**, goals .53**, impulse .46**, awareness .34**, strategies .69**, clarity .39**) and the measure of experiential avoidance although only 3 subscales were sig correlated with emotional expressivity (nonacceptance, awareness, clarity) |
| **Personality** | **Eating Disorder Inventory**  **Drive for thinness subscale** and  **Perfectionism subscale item**  Garner^24^ | **Cronbach's α**= 0.85 for drive for thinness in AN group, α =0.82 for perfectionism in AN group  **Item-total correlation** for Perfectionism subscale item 5, "I feel I must do perfectly or not do them at all", α =0.63 | **Construct validity: "**congruence between clinician ratings (see below) and patients subscale provides some evidence of construct validity**"**  **Criterion validity:** correlated AN patients' subscale scores with clinicians' ratings (drive for thinness correlation 0.53, % of AN and female control groups correctly classified 89.9%; Perfectionism subscale r=.47, 89.2%)**;** correlation drive for thinness and EAT ('anorexic' attitudes scale by Garner and Garfinkel 1979) r=.88 p<.001 Restraint scale (Harvy &Polivy) r=.50 p<.001  **Convergent and discriminant validity:**  'established for all subscales' |
| **Personality** | Single-item **Self-Esteem** Scale (SISE)  Robins et al.^25^ | In 3 studies, **disattenuated correlations** between the SISE and the Rosenberg (1965) Self-Esteem (RSE) scale approached 1.00 (disattenuated correlations ranged from .91 to .99, whereas **correlations** ranged from .74 to .80).^25^  Since **Cronbach’s α** not applicable as it cannot be computed for single item, they used the **Heise procedure** which estimates reliability of a single-item scale based on its pattern of autocorrelations over 3 time points, hence provides a measure of **test-retest reliability:** the mean reliability (3 tests) was 0.75. (they compared this to Heise for RSE which was 0.88) To examine stability over time, *r* of the SISE and RSE were computed across 6 assessments. The mean across-time correlation (averaged across all 15 possible time intervals) was similar for the SISE (mean *r*=.61) and the RSE (mean *r* = .69). | **Convergent** reliability: “*strong*” convergence with Rosenberg Self-Esteem scale.  Factor analysis performed, suggested a single general factor: all items had high loadings on the 1^st^ unrotated factor, a screen test showed an “elbow“ after the first factor and an analysis using structural equation modeling showed relatively good fit with a single factor model, comparative fit index= 0.90)  Convergent and discriminant validity also performed across 37 different criteria, “the two measures showed remarkably similar correlations with the domain-specific self-evaluations“ |
| **Physical determinants** | **Pre-pregnancy BMI,**  **Number of pregnancies** | Standard questions routinely asked of patients during every pregnancy | Not applicable as standard questions |
| **Lifestyle determinants** | **Smoking** | Standard questions routinely asked of patients during every pregnancy | Not applicable as standard questions |
| **Lifestyle determinants** | **Physical activity scale**  **Screen time and sleep time**  Aadahl^26^ | **Test-retest** intraclass correlation coefficient within 2 weeks = 0.87 ^27^  Wilcoxon Test *P* = 0.72 | **Criterion validity**: significantly positively correlated with VO_2max_^28^ |
| **Lifestyle determinants** | **Kaiser Physical Activity Questionnaire (validated in pregnant women)**  Schmidt et al.^29^ | **Test-retest** intraclass correlation coefficient after 7 days=  Total Activity: 0.84; total weighted activity 0.76; Household/caregiving: 0.85  Occupational: 0.86; Active living: 0.76  Sports/exercise: 0.84 | **Criterion validity**: **has** **highest correlation with accelerometer of any self-reported scale in pregnancy**: Spearman correlation coefficients (average counts per minute) Total activity: 0.52; total weighted activity 0.59  By type: Household/caregiving: 0.23, Occupational: 0.25  Active living: 0.32, Sports/exercise: 0.40; **Correlation with VO_2max_**=0.62 for 3-point summary index^30^ |
| **Lifestyle determinants** | **Food frequency questionnaire** [FFQ], Block-60 item  Block^31^ | **Cronbach’s α** and test-retest correlation not mentioned. | Since most of variance in intake can be explained by a list of 20 foods^32^, we chose the reduced (to 60) item FFQ rather than the 100-item. **Correlations between Block-60 item and Block--100 item were all above 0.94.**  **Validated against 4-d records**, 36.1% fat vs 37.7% in record, 1142 cal vs 1560 in record, p<0.01; like all FFQs, there are differences with records, therefore 24-hr record will also be used. |
| **Pregnancy Symptoms** | **RBWH Pregnancy Symptoms Inventory,** for nausea.  Foxcroft et al.^33^ | No validated scales for other questions on pregnancy symptoms but inserted as per Reviewer 2’s important suggestion |  |

Abbreviations: α = alpha, AN = anorexia nervosa, β = beta, BIA-O = Body Image Assessment for Obesity, BMI = body mass index, BSQ = Body Shape Questionnaire, CBS = current body size, DERS = Difficulties in Emotion Regulation, DIS = Disinhibitive Eating Scale, EAT = Eating Attitudes Test, EDEQ = Eating Disorders Examination Questionnaire, IBS = ideal body size, NMR = Negative Mood Regulation, ρ = Spearman's rank correlation coefficient, PHQ = Patient Health Questionnaire, *r* = Pearson product-moment correlation coefficient, RSE = Rosenberg Self-Esteem, RBWH = Royal Brisbane and Women’s Hospital, SAS = Statistical Analytic System (software provided by SAS Institute Inc., Cary, NC), SE = standard error, SEM = standard error of the mean, SISE = Single-Item Self-Esteem, SMI-10 = Self-Motivation Inventory 10-item short form scale, TIPI = Ten Item Personality Inventory.

Reference List

(1) van Strien T, Frijters JER, Bergers GPA, Defares PB. The Dutch Eating Behavior Questionnaire (DEBQ) for assessment of restrained, emotional, and external eating behavior. *Int J Eat Disord* 1986; 5:295-315. doi: 10.1002/1098-108X(198602)5:2<295::AID-EAT2260050209>3.0.CO;2-T.

(2) Herman CP, Polivy J, Pliner P, Threlkeld J, Munic D. Distractibility in dieters and nondieters: an alternative view of "externality". *J Pers Soc Psychol* 1978; 36(5):536-548.

(3) van Strien T, Breteler M, Ouwens M. Restraint Scale, its sub-scales concern for dieting and weight fluctuation. *Pers Individ Dif* 2002; 33(5):791-802.

(4) Overduin J, Jansen A. A new scale for use in non-clinical research into disinhibitive eating. *Pers Individ Dif* 1996; 20(6):669-677.

(5) Hales D, Evenson KR, Wen F, Wilcox S. Postpartum physical activity: measuring theory of planned behavior constructs. *Am J Health Behav* 2010; 34(4):387-401.

(6) Fowles ER, Feucht J. Testing the barriers to healthy eating scale. *West J Nurs Res* 2004; 26(4):429-443.

(7) Kendall A, Olson CM, Frongillo EA, Jr. Evaluation of psychosocial measures for understanding weight-related behaviors in pregnant women. *Ann Behav Med* 2001; 23(1):50-58.

(8) Saltzer EB. The weight locus of control (WLOC) scale: a specific measure for obesity research. *J Pers Assess* 1982; 46(6):620-628.

(9) Palmer JL, Jennings GE, Massey L. Development of an assessment form: attitude toward weight gain during pregnancy. *J Am Diet Assoc* 1985; 85(8):946-949.

(10) Dishman RK, Ickes W. Self-motivation and adherence to therapeutic exercise. *J Behav Med* 1981; 4(4):421-438.

(11) Teixeira PJ, Going SB, Houtkooper LB, Cussler EC, Martin CJ, Metcalfe LL et al. Weight loss readiness in middle-aged women: psychosocial predictors of success for behavioral weight reduction. *J Behav Med* 2002; 25(6):499-523.

(12) Williams GC, Grow VM, Freedman ZR, Ryan RM, Deci EL. Motivational predictors of weight loss and weight-loss maintenance. *J Pers Soc Psychol* 1996; 70(1):115-126.

(13) Parmenter K, Wardle J. Development of a general nutrition knowledge questionnaire for adults. *Eur J Clin Nutr* 1999; 53(4):298-308.

(14) Williamson DA, Womble LG, Zucker NL, Reas DL, White MA, Blouin DC et al. Body image assessment for obesity (BIA-O): development of a new procedure. *Int J Obes Relat Metab Disord* 2000; 24(10):1326-1332.

(15) Cogswell ME, Scanlon KS, Fein SB, Schieve LA. Medically advised, mother's personal target, and actual weight gain during pregnancy. *Obstet Gynecol* 1999; 94(4):616-622.

(16) Kroenke K, Spitzer RL, Williams JB, Lowe B. An ultra-brief screening scale for anxiety and depression: the PHQ-4. *Psychosomatics* 2009; 50(6):613-621.

(17) Kroenke K, Spitzer RL, Williams JB. The Patient Health Questionnaire-2: validity of a two-item depression screener. *Med Care* 2003; 41(11):1284-1292.

(18) Kroenke K, Spitzer RL, Williams JB, Monahan PO, Lowe B. Anxiety disorders in primary care: prevalence, impairment, comorbidity, and detection. *Ann Intern Med* 2007; 146(5):317-325.

(19) Cox JL, Holden JM, Sagovsky R. Detection of postnatal depression. Development of the 10-item Edinburgh Postnatal Depression Scale. *Br J Psychiatry* 1987; 150:782-786.

(20) Rini CK, Dunkel-Schetter C, Wadhwa PD, Sandman CA. Psychological adaptation and birth outcomes: the role of personal resources, stress, and sociocultural context in pregnancy. *Health Psychol* 1999; 18(4):333-345.

(21) Gosling SD, Rentfrow PJ, Swann WB Jr. A very brief measure of the Big-Five personality domains. *Journal of Research in Personality* 2003; 37:504-528.

(22) Gross JJ, John OP. Individual differences in two emotion regulation processes: implications for affect, relationships, and well-being. *J Pers Soc Psychol* 2003; 85(2):348-362.

(23) Gratz K, Roemer L. Multidimensional assessment of emotion regulation and dysregulation: Development, factor structure, and initial validation of the difficulties in emotion regulation scale. *J Psychopathol Behav Assess* 2004; 26(1):41-54.

(24) Garner D, Olmstead M, Polivy J. Development and validation of a multidimensional eating disorder inventory for anorexia nervosa and bulimia. *Int J Eat Disord* 1983; 2(2):15-34.

(25) Robins RW, Hendin HM, Trzesniewski KH. Measuring global self-esteem: Construct validation of a single-item measure and the Rosenburg Self-Esteem Scale. *Personality and Social Psychology Bulletin* 2001; 27(2):151-161.

(26) Aadahl M, Jorgensen T. Validation of a new self-report instrument for measuring physical activity. *Med Sci Sports Exerc* 2003; 35(7):1196-1202.

(27) Jiang H, He G, Li M, Fan Y, Jiang H, Bauman A et al. Reliability and Validity of a Physical Activity Scale Among Urban Pregnant Women in Eastern China. *Asia Pac J Public Health* 2012.

(28) Aadahl M, Kjaer M, Kristensen JH, Mollerup B, Jorgensen T. Self-reported physical activity compared with maximal oxygen uptake in adults. *Eur J Cardiovasc Prev Rehabil* 2007; 14(3):422-428.

(29) Schmidt MD, Freedson PS, Pekow P, Roberts D, Sternfeld B, Chasan-Taber L. Validation of the Kaiser Physical Activity Survey in pregnant women. *Med Sci Sports Exerc* 2006; 38(1):42-50.

(30) Ainsworth BE, Sternfeld B, Richardson MT, Jackson K. Evaluation of the kaiser physical activity survey in women. *Med Sci Sports Exerc* 2000; 32(7):1327-1338.

(31) Block G, Hartman AM, Naughton D. A reduced dietary questionnaire: development and validation. *Epidemiology* 1990; 1(1):58-64.

(32) Byers T, Marshall J, Fiedler R, Zielezny M, Graham S. Assessing nutrient intake with an abbreviated dietary interview. *Am J Epidemiol* 1985; 122(1):41-50.

(33) Foxcroft KF, Callaway LK, Byrne NM, Webster J. Development and validation of a pregnancy symptoms inventory. *BMC Pregnancy Childbirth* 2013; 13:3.
