# Supplementary material for: Gestational weight gain outside the 2009 Institute of Medicine recommendations: novel psychological and behavioural factors associated with inadequate or excess weight gain in a prospective cohort study
Source: BMC Pregnancy Childbirth. 2021 Jan 21;21:70. doi: 10.1186/s12884-021-03555-5 (PMC7818557; doi:10.1186/s12884-021-03555-5)
Supplement: Supplementary file 3 — Additional file 3. Univariable multinomial logistic regression analyses assessing associations between exposure variables with inadequate or excessive gestational weight gain in a prospective cohort study on predictors of guideline-discordant gestational weight gain. Table of univariable multinomial logistic regression analyses assessing associations between exposure variables with inadequate or excessive gestational weight gain in a prospective cohort study on predictors of guideline-discordant gestational weight gain. [file 12884_2021_3555_MOESM3_ESM.docx]

| **Variable/exposure** | **n** | **Reference** | **Outcome** | **OR (95% CI)** | ***p-value* for each category** | **Overall *p-value*** |
| --- | --- | --- | --- | --- | --- | --- |
| **Maternal age, yr** | 970 |  |  |  |  | 0.098 |
| Continuous |  | Continuous | Inadequate GWG | 1.00 (0.96 to 1.04) | 0.891 |  |
| Continuous |  | Continuous | Excess GWG | 0.97 (0.94 to 1.00) | 0.051 |  |
| **Race** | 967 |  |  |  |  | 0.011 |
| Non-white |  | White | Inadequate GWG | 0.97 (0.63 to 1.51) | 0.900 |  |
| Non-white |  | White | Excess GWG | 0.63 (0.45 to 0.88) | 0.007 |  |
| **Marital status** | 968 |  |  |  |  | 0.997 |
| Single, divorced, widowed |  | Married, common-law, living w/ a partner | Inadequate GWG | 1.00 (0.46 to 2.14) | 0.992 |  |
| Single, divorced, widowed |  | Married, common-law, living w/ a partner | Excess GWG | 1.02 (0.58 to 1.78) | 0.950 |  |
| **Education** | 969 |  |  |  |  | 0.593 |
| Undergraduate university or higher |  | Community college/technical school or lower | Inadequate GWG | 0.83 (0.56 to 1.23) | 0.347 |  |
| Undergraduate university or higher |  | Community college/technical school or lower | Excess GWG | 0.89 (0.66 to 1.19) | 0.418 |  |
| **Household income** | 877 |  |  |  |  | 0.451 |
| $40,000 – 79,999 |  | < $40,000 | Inadequate GWG | 0.98 (0.53 to 1.82) | 0.947 |  |
| $40,000 – 79,999 |  | < $40,000 | Excess GWG | 1.31 (0.82 to 2.09) | 0.262 |  |
| ≥$80,000 |  | < $40,000 | Inadequate GWG | 0.81 (0.47 to 1.40) | 0.448 |  |
| ≥$80,000 |  | < $40,000 | Excess GWG | 0.96 (0.63 to 1.46) | 0.847 |  |
| **Smoking** | 968 |  |  |  |  | 0.017 |
| Before this pregnancy |  | Never | Inadequate GWG | 0.82 (0.40 to 1.67) | 0.576 |  |
| Before this pregnancy |  | Never | Excess GWG | 1.79 (1.13 to 2.84) | 0.014 |  |
| During this pregnancy |  | Never | Inadequate GWG | 1.71 (0.80 to 3.66) | 0.165 |  |
| During this pregnancy |  | Never | Excess GWG | 1.44 (0.77 to 2.67) | 0.250 |  |
| **Parity** | 965 |  |  |  |  | 0.115 |
| 1+ children |  | None | Inadequate GWG | 1.12 (0.75 to 1.66) | 0.584 |  |
| 1+ children |  | None | Excess GWG | 0.80 (0.60 to 1.07) | 0.136 |  |
| **Prepregnancy BMI** | 970 |  |  |  |  | <0.001 |
| Underweight |  | Normal weight | Inadequate GWG | 0.69 (0.26 to 1.83) | 0.456 |  |
| Underweight |  | Normal weight | Excess GWG | 0.43 (0.18 to 1.03) | 0.057 |  |
| Overweight |  | Normal weight | Inadequate GWG | 0.37 (0.18 to 0.77) | 0.008 |  |
| Overweight |  | Normal weight | Excess GWG | 3.05 (2.09 to 4.47) | <0.001 |  |
| Obese |  | Normal weight | Inadequate GWG | 1.45 (0.88 to 2.39) | 0.147 |  |
| Obese |  | Normal weight | Excess GWG | 2.45 (1.65 to 3.63) | <0.001 |  |
| **Depression** | 970 |  |  |  |  | 0.029 |
| Yes |  | No | Inadequate GWG | 2.77 (1.15 to 6.63) | 0.023 |  |
| Yes |  | No | Excess GWG | 2.61 (1.25 to 5.44) | 0.010 |  |
| **Anxiety** | 970 |  |  |  |  | 0.001 |
| Yes |  | No | Inadequate GWG | 3.28 (1.72 to 6.23) | <0.001 |  |
| Yes |  | No | Excess GWG | 2.01 (1.15 to 3.51) | 0.014 |  |
| **Other chronic health conditions** | 970 |  |  |  |  | 0.685 |
| Other chronic health conditions, yes |  | None | Inadequate GWG | 1.21 (0.78 to 1.88) | 0.396 |  |
| Other chronic health conditions, yes |  | None | Excess GWG | 1.10 (0.79 to 1.54) | 0.555 |  |
| **Satisfied with weight before pregnancy** | 962 |  |  |  |  | 0.001 |
| Somewhat satisfied or very satisfied |  | Not satisfied at all or not very satisfied | Inadequate GWG | 0.81 (0.53 to 1.25) | 0.339 |  |
| Somewhat satisfied or very satisfied |  | Not satisfied at all or not very satisfied | Excess GWG | 0.57 (0.41 to 0.78) | 0.000 |  |
| **Planned total gestational weight gain** | 970 |  |  |  |  | <0.001 |
| None |  | Within IOM guidelines | Inadequate GWG | 3.45 (1.57 to 7.58) | 0.002 |  |
| None |  | Within IOM guidelines | Excess GWG | 1.73 (0.89 to 3.37) | 0.105 |  |
| Below IOM guidelines |  | Within IOM guidelines | Inadequate GWG | 2.29 (1.43 to 3.65) | 0.001 |  |
| Below IOM guidelines |  | Within IOM guidelines | Excess GWG | 0.89 (0.62 to 1.28) | 0.538 |  |
| Above IOM guidelines |  | Within IOM guidelines | Inadequate GWG | 1.19 (0.65 to 2.16) | 0.575 |  |
| Above IOM guidelines |  | Within IOM guidelines | Excess GWG | 2.88 (1.98 to 4.19) | 0.000 |  |
| **Recommended weight gain levels by healthcare provider** | 970 |  |  |  |  | 0.295 |
| None |  | Within IOM guidelines | Inadequate GWG | 0.94 (0.49 to 1.80) | 0.849 |  |
| None |  | Within IOM guidelines | Excess GWG | 1.42 (0.85 to 2.38) | 0.182 |  |
| Below IOM guidelines |  | Within IOM guidelines | Inadequate GWG | 2.25 (0.70 to 7.20) | 0.172 |  |
| Below IOM guidelines |  | Within IOM guidelines | Excess GWG | 1.58 (0.57 to 4.38) | 0.381 |  |
| Above IOM guidelines |  | Within IOM guidelines | Inadequate GWG | 0.58 (0.14 to 2.47) | 0.464 |  |
| Above IOM guidelines |  | Within IOM guidelines | Excess GWG | 1.96 (0.79 to 4.87) | 0.145 |  |
| Not reported/I cannot remember |  | Within IOM guidelines | Inadequate GWG | 0.93 (0.32 to 2.68) | 0.898 |  |
| Not reported/I cannot remember |  | Within IOM guidelines | Excess GWG | 1.77 (0.81 to 3.84) | 0.150 |  |
| **Perceived weight gain recommendation for the 1^st^ trimester** | 970 |  |  |  |  | 0.399 |
| None |  | Within IOM guidelines | Inadequate GWG | 1.66 (0.55 to 5.02) | 0.368 |  |
| None |  | Within IOM guidelines | Excess GWG | 1.42 (0.57 to 3.57) | 0.456 |  |
| Outside IOM guidelines |  | Within IOM guidelines | Inadequate GWG | 0.85 (0.56 to 1.31) | 0.463 |  |
| Outside IOM guidelines |  | Within IOM guidelines | Excess GWG | 1.17 (0.85 to 1.61) | 0.342 |  |
| **Do you believe that there are any risks to you by gaining too little weight during pregnancy?** | 964 |  |  |  |  | 0.567 |
| Yes |  | No (too little GWG, risk for the mom) | Inadequate GWG | 1.09 (0.73 to 1.63) | 0.678 |  |
| Yes |  | No (too little GWG, risk for the mom) | Excess GWG | 0.91 (0.67 to 1.22) | 0.510 |  |
| **Do you believe that there are any risks to the baby by you gaining too little weight during pregnancy?** | 963 |  |  |  |  | 0.705 |
| Yes |  | No (too little GWG, risk for the baby) | Inadequate GWG | 1.14 (0.69 to 1.89) | 0.610 |  |
| Yes |  | No (too little GWG, risk for the baby) | Excess GWG | 0.94 (0.66 to 1.35) | 0.734 |  |
| **Do you believe that there any risks for you to gaining too much weight during pregnancy?** | 963 |  |  |  |  | 0.841 |
| Yes |  | No (too much GWG, risk for the mom) | Inadequate GWG | 0.80 (0.36 to 1.76) | 0.575 |  |
| Yes |  | No (too much GWG, risk for the mom) | Excess GWG | 0.87 (0.47 to 1.60) | 0.656 |  |
| **Do you believe that there are any risks to the baby by you gaining too much weight during pregnancy?** | 962 |  |  |  |  | 0.743 |
| Yes |  | No (too much GWG, risk for the baby) | Inadequate GWG | 1.30 (0.67 to 2.51) | 0.441 |  |
| Yes |  | No (too much GWG, risk for the baby) | Excess GWG | 1.09 (0.69 to 1.72) | 0.718 |  |
| **Whether my weight changes is up to me** | 961 |  |  |  |  | 0.820 |
| Disagree or strongly disagree |  | Neither disagree nor agree | Inadequate GWG | 0.96 (0.57 to 1.59) | 0.861 |  |
| Disagree or strongly disagree |  | Neither disagree nor agree | Excess GWG | 1.16 (0.79 to 1.69) | 0.449 |  |
| Agree or strongly agree |  | Neither disagree nor agree | Inadequate GWG | 0.88 (0.54 to 1.43) | 0.602 |  |
| Agree or strongly agree |  | Neither disagree nor agree | Excess GWG | 0.97 (0.68 to 1.40) | 0.882 |  |
| **If I eat right, and can get enough exercise and rest, I can control my weight the way I want** | 965 |  |  |  |  | 0.109 |
| Disagree or strongly disagree |  | Neither disagree nor agree | Inadequate GWG | 1.47 (0.77 to 2.82) | 0.247 |  |
| Disagree or strongly disagree |  | Neither disagree nor agree | Excess GWG | 1.21 (0.73 to 2.02) | 0.460 |  |
| Agree or strongly agree |  | Neither disagree nor agree | Inadequate GWG | 0.75 (0.45 to 1.27) | 0.287 |  |
| Agree or strongly agree |  | Neither disagree nor agree | Excess GWG | 0.78 (0.53 to 1.15) | 0.211 |  |
| **Being the right weight is mainly good luck** | 959 |  |  |  |  | 0.998 |
| Agree or strongly agree |  | Neither disagree nor agree | Inadequate GWG | 0.98 (0.42 to 2.26) | 0.959 |  |
| Agree or strongly agree |  | Neither disagree nor agree | Excess GWG | 0.91 (0.49 to 1.69) | 0.775 |  |
| Disagree or strongly disagree |  | Neither disagree nor agree | Inadequate GWG | 0.98 (0.59 to 1.64) | 0.943 |  |
| Disagree or strongly disagree |  | Neither disagree nor agree | Excess GWG | 0.94 (0.65 to 1.37) | 0.755 |  |
| **You can’t control the amount of weight you gain when you are pregnant** | 963 |  |  |  |  | 0.313 |
| Agree or strongly agree |  | Neither disagree nor agree | Inadequate GWG | 1.13 (0.55 to 2.32) | 0.743 |  |
| Agree or strongly agree |  | Neither disagree nor agree | Excess GWG | 1.30 (0.78 to 2.17) | 0.320 |  |
| Disagree or strongly disagree |  | Neither disagree nor agree | Inadequate GWG | 1.06 (0.64 to 1.75) | 0.830 |  |
| Disagree or strongly disagree |  | Neither disagree nor agree | Excess GWG | 0.85 (0.59 to 1.23) | 0.389 |  |
| **Think that family and friends believe that pregnant women need to eat two times as much as before pregnancy** | 966 |  |  |  |  | 0.250 |
| Disagree or strongly disagree |  | Neither disagree nor agree | Inadequate GWG | 1.22 (0.65 to 2.30) | 0.541 |  |
| Disagree or strongly disagree |  | Neither disagree nor agree | Excess GWG | 1.62 (1.00 to 2.60) | 0.048 |  |
| Agree or strongly agree |  | Neither disagree nor agree | Inadequate GWG | 1.48 (0.66 to 3.33) | 0.342 |  |
| Agree or strongly agree |  | Neither disagree nor agree | Excess GWG | 2.00 (1.08 to 3.68) | 0.027 |  |
| **Think that family and friends believe that pregnant women crave foods more intensely than other people** | 963 |  |  |  |  | 0.188 |
| Disagree or strongly disagree |  | Neither disagree nor agree | Inadequate GWG | 1.82 (0.87 to 3.80) | 0.114 |  |
| Disagree or strongly disagree |  | Neither disagree nor agree | Excess GWG | 0.84 (0.48 to 1.49) | 0.550 |  |
| Agree or strongly agree |  | Neither disagree nor agree | Inadequate GWG | 1.12 (0.63 to 1.97) | 0.707 |  |
| Agree or strongly agree |  | Neither disagree nor agree | Excess GWG | 0.87 (0.59 to 1.30) | 0.502 |  |
| **Think that family and friends believe that pregnant women should eat what they crave** | 965 |  |  |  |  | 0.926 |
| Disagree or strongly disagree |  | Neither disagree nor agree | Inadequate GWG | 0.97 (0.61 to 1.56) | 0.907 |  |
| Disagree or strongly disagree |  | Neither disagree nor agree | Excess GWG | 1.07 (0.75 to 1.51) | 0.723 |  |
| Agree or strongly agree |  | Neither disagree nor agree | Inadequate GWG | 0.83 (0.51 to 1.34) | 0.440 |  |
| Agree or strongly agree |  | Neither disagree nor agree | Excess GWG | 0.96 (0.68 to 1.37) | 0.839 |  |
| **Think that family and friends believe that pregnant women should not exert themselves physically** | 961 |  |  |  |  | 0.058 |
| Disagree or strongly disagree |  | Neither disagree nor agree | Inadequate GWG | 1.69 (0.92 to 3.11) | 0.088 |  |
| Disagree or strongly disagree |  | Neither disagree nor agree | Excess GWG | 1.09 (0.72 to 1.66) | 0.672 |  |
| Agree or strongly agree |  | Neither disagree nor agree | Inadequate GWG | 1.27 (0.71 to 2.27) | 0.426 |  |
| Agree or strongly agree |  | Neither disagree nor agree | Excess GWG | 0.74 (0.50 to 1.10) | 0.133 |  |
| **Think that family and friends believe that pregnant women should not be worried about gaining too much weight during pregnancy** | 962 |  |  |  |  | 0.331 |
| Disagree or strongly disagree |  | Neither disagree nor agree | Inadequate GWG | 1.13 (0.66 to 1.93) | 0.667 |  |
| Disagree or strongly disagree |  | Neither disagree nor agree | Excess GWG | 1.09 (0.74 to 1.60) | 0.665 |  |
| Agree or strongly agree |  | Neither disagree nor agree | Inadequate GWG | 1.61 (0.90 to 2.89) | 0.108 |  |
| Agree or strongly agree |  | Neither disagree nor agree | Excess GWG | 1.44 (0.94 to 2.21) | 0.094 |  |
| **How often do you eat meals in front of a screen?** | 956 |  |  |  |  | <0.001 |
| Some meals |  | None or almost no meals | Inadequate GWG | 1.22 (0.78 to 1.89) | 0.379 |  |
| Some meals |  | None or almost no meals | Excess GWG | 1.99 (1.44 to 2.75) | <0.001 |  |
| Most meals or more |  | None or almost no meals | Inadequate GWG | 1.72 (0.98 to 3.04) | 0.061 |  |
| Most meals or more |  | None or almost no meals | Excess GWG | 1.88 (1.20 to 2.93) | 0.005 |  |
| **How often do you watch television before going to sleep?** | 959 |  |  |  |  | 0.578 |
| Some nights |  | None or almost no meals | Inadequate GWG | 1.11 (0.64 to 1.91) | 0.715 |  |
| Some nights |  | None or almost no meals | Excess GWG | 1.32 (0.89 to 1.96) | 0.167 |  |
| Most nights or more |  | None or almost no meals | Inadequate GWG | 1.28 (0.77 to 2.11) | 0.339 |  |
| Most nights or more |  | None or almost no meals | Excess GWG | 1.18 (0.80 to 1.72) | 0.403 |  |
| **During a typical day, do you drink soda pop, cola, or juice?** | 968 |  |  |  |  | 0.745 |
| Yes |  | No | Inadequate GWG | 0.94 (0.63 to 1.40) | 0.767 |  |
| Yes |  | No | Excess GWG | 1.07 (0.80 to 1.44) | 0.634 |  |
| **On average, how many times would you eat fast food?** | 968 |  |  |  |  | 0.058 |
| 2 or 3 times per month |  | <= 1 time per month | Inadequate GWG | 0.71 (0.44 to 1.14) | 0.156 |  |
| 2 or 3 times per month |  | <= 1 time per month | Excess GWG | 1.27 (0.89 to 1.81) | 0.180 |  |
| ≥1 time per week |  | <= 1 time per month | Inadequate GWG | 0.84 (0.52 to 1.36) | 0.486 |  |
| ≥1 time per week |  | <= 1 time per month | Excess GWG | 1.37 (0.95 to 1.97) | 0.094 |  |
| **Fruit and vegetable intake per day** | 955 |  |  |  |  | 0.835 |
| ≥ 5 servings/day |  | < 5 servings/day | Inadequate GWG | 0.90 (0.60 to 1.35) | 0.618 |  |
| ≥ 5 servings/day |  | < 5 servings/day | Excess GWG | 1.01 (0.75 to 1.35) | 0.956 |  |
| **On average, how many times would you eat snack foods?** | 967 |  |  |  |  | 0.563 |
| ≥ 2 times per week |  | <= 1 time per week | Inadequate GWG | 1.05 (0.70 to 1.56) | 0.821 |  |
| ≥ 2 times per week |  | <= 1 time per week | Excess GWG | 1.17 (0.87 to 1.56) | 0.305 |  |
| **How much of your daily food intake do you eat after suppertime?** | 966 |  |  |  |  | 0.947 |
| ≥ 1/4 |  | < 1/4 | Inadequate GWG | 0.90 (0.41 to 1.96) | 0.782 |  |
| ≥ 1/4 |  | < 1/4 | Excess GWG | 0.92 (0.52 to 1.62) | 0.772 |  |
| **Do you snack in the middle of the night?** | 963 |  |  |  |  | 0.337 |
| Yes |  | No | Inadequate GWG | 1.54 (0.86 to 2.78) | 0.148 |  |
| Yes |  | No | Excess GWG | 1.27 (0.80 to 2.02) | 0.302 |  |
| **During this pregnancy, do you have feelings of guilt after overeating?** | 939 |  |  |  |  | 0.572 |
| Often or always |  | Never or rarely | Inadequate GWG | 1.00 (0.57 to 1.74) | 0.992 |  |
| Often or always |  | Never or rarely | Excess GWG | 1.20 (0.81 to 1.79) | 0.361 |  |
| **During this pregnancy, do you ever feel that when you started eating you just couldn’t stop?** | 941 |  |  |  |  | 0.263 |
| Often or always |  | Never or rarely | Inadequate GWG | 1.27 (0.61 to 2.65) | 0.523 |  |
| Often or always |  | Never or rarely | Excess GWG | 1.56 (0.91 to 2.69) | 0.108 |  |
| **During pregnancy, you can eat foods that are good for you even when family or social life takes a lot of your time** | 965 |  |  |  |  | 0.545 |
| Unsure or very unsure |  | Neither unsure nor sure | Inadequate GWG | 0.84 (0.27 to 2.63) | 0.760 |  |
| Unsure or very unsure |  | Neither unsure nor sure | Excess GWG | 1.19 (0.57 to 2.48) | 0.647 |  |
| Sure or very sure |  | Neither unsure nor sure | Inadequate GWG | 1.29 (0.65 to 2.56) | 0.470 |  |
| Sure or very sure |  | Neither unsure nor sure | Excess GWG | 0.95 (0.59 to 1.52) | 0.819 |  |
| **During pregnancy, you can get regular exercise** | 965 |  |  |  |  | 0.194 |
| Unsure or very unsure |  | Neither unsure nor sure | Inadequate GWG | 2.44 (1.06 to 5.62) | 0.036 |  |
| Unsure or very unsure |  | Neither unsure nor sure | Excess GWG | 1.07 (0.59 to 1.93) | 0.829 |  |
| Sure or very sure |  | Neither unsure nor sure | Inadequate GWG | 1.54 (0.79 to 3.01) | 0.210 |  |
| Sure or very sure |  | Neither unsure nor sure | Excess GWG | 0.88 (0.57 to 1.34) | 0.547 |  |
| **I control my emotions by not expressing them** | 968 |  |  |  |  | 0.230 |
| Almost never or sometimes |  | Most of the time or almost always | Inadequate GWG | 0.91 (0.44 to 1.91) | 0.808 |  |
| Almost never or sometimes |  | Most of the time or almost always | Excess GWG | 1.00 (0.58 to 1.72) | 0.995 |  |
| About half the time |  | Most of the time or almost always | Inadequate GWG | 1.56 (0.67 to 3.61) | 0.300 |  |
| About half the time |  | Most of the time or almost always | Excess GWG | 1.02 (0.54 to 1.94) | 0.948 |  |
| **When I am upset, I have difficulty controlling my behaviour** | 958 |  |  |  |  | 0.995 |
| Almost never or sometimes |  | Most of the time or almost always | Inadequate GWG | 1.09 (0.47 to 2.49) | 0.846 |  |
| Almost never or sometimes |  | Most of the time or almost always | Excess GWG | 1.05 (0.58 to 1.90) | 0.875 |  |
| About half the time |  | Most of the time or almost always | Inadequate GWG | 1.20 (0.43 to 3.34) | 0.727 |  |
| About half the time |  | Most of the time or almost always | Excess GWG | 1.03 (0.48 to 2.17) | 0.948 |  |
| **When I’m upset, it takes me a long time to feel better** | 958 |  |  |  |  | 0.462 |
| Almost never or sometimes |  | Most of the time or almost always | Inadequate GWG | 0.76 (0.34 to 1.70) | 0.508 |  |
| Almost never or sometimes |  | Most of the time or almost always | Excess GWG | 0.64 (0.36 to 1.16) | 0.142 |  |
| About half the time |  | Most of the time or almost always | Inadequate GWG | 1.03 (0.41 to 2.60) | 0.955 |  |
| About half the time |  | Most of the time or almost always | Excess GWG | 0.65 (0.32 to 1.31) | 0.231 |  |
| **When I’m upset, I believe that there’s nothing I can do to make myself feel better** | 967 |  |  |  |  | 0.450 |
| Almost never or sometimes |  | Most of the time or almost always | Inadequate GWG | 0.57 (0.14 to 2.32) | 0.433 |  |
| Almost never or sometimes |  | Most of the time or almost always | Excess GWG | 0.98 (0.29 to 3.28) | 0.972 |  |
| About half the time |  | Most of the time or almost always | Inadequate GWG | 0.25 (0.05 to 1.36) | 0.109 |  |
| About half the time |  | Most of the time or almost always | Excess GWG | 0.75 (0.20 to 2.83) | 0.671 |  |
| **When I'm upset, I know I can find a way to eventually feel better** | 965 |  |  |  |  | 0.250 |
| Most of the time or almost always |  | Almost never or sometimes | Inadequate GWG | 0.48 (0.25 to 0.94) | 0.031 |  |
| Most of the time or almost always |  | Almost never or sometimes | Excess GWG | 0.66 (0.38 to 1.15) | 0.144 |  |
| About half the time |  | Almost never or sometimes | Inadequate GWG | 0.56 (0.22 to 1.42) | 0.223 |  |
| About half the time |  | Almost never or sometimes | Excess GWG | 0.85 (0.41 to 1.75) | 0.654 |  |
| **When I am upset, I become embarrassed for feeling that way** | 965 |  |  |  |  | 0.689 |
| Almost never or sometimes |  | Most of the time or almost always | Inadequate GWG | 0.60 (0.29 to 1.21) | 0.153 |  |
| Almost never or sometimes |  | Most of the time or almost always | Excess GWG | 0.81 (0.45 to 1.43) | 0.464 |  |
| About half the time |  | Most of the time or almost always | Inadequate GWG | 0.56 (0.23 to 1.40) | 0.218 |  |
| About half the time |  | Most of the time or almost always | Excess GWG | 0.84 (0.42 to 1.71) | 0.635 |  |
| **I feel that I must do things perfectly or not do them at all** | 964 |  |  |  |  | 0.158 |
| Almost never or sometimes |  | Most of the time or almost always | Inadequate GWG | 0.85 (0.47 to 1.52) | 0.581 |  |
| Almost never or sometimes |  | Most of the time or almost always | Excess GWG | 0.85 (0.54 to 1.34) | 0.483 |  |
| About half the time |  | Most of the time or almost always | Inadequate GWG | 0.36 (0.15 to 0.88) | 0.024 |  |
| About half the time |  | Most of the time or almost always | Excess GWG | 0.83 (0.47 to 1.47) | 0.530 |  |
| **During the 3 months before pregnancy, how often were you dieting?** | 968 |  |  |  |  | 0.007 |
| Often or always |  | Never or rarely | Inadequate GWG | 0.96 (0.56 to 1.67) | 0.895 |  |
| Often or always |  | Never or rarely | Excess GWG | 1.67 (1.14 to 2.45) | 0.008 |  |
| **During the 3 months before pregnancy, how often did you limit your carbohydrate and sugar intake?** | 969 |  |  |  |  | 0.033 |
| Often or always |  | Never or rarely | Inadequate GWG | 0.75 (0.50 to 1.13) | 0.171 |  |
| Often or always |  | Never or rarely | Excess GWG | 1.22 (0.91 to 1.63) | 0.189 |  |
| **During the 3 months before pregnancy, did you have feelings of guilt after overeating?** | 969 |  |  |  |  | 0.027 |
| Often or always |  | Never or rarely | Inadequate GWG | 1.10 (0.71 to 1.71) | 0.660 |  |
| Often or always |  | Never or rarely | Excess GWG | 1.50 (1.09 to 2.06) | 0.012 |  |
| **During the 3 months before pregnancy, did you ever feel that when you started eating you just couldn’t stop?** | 968 |  |  |  |  | 0.047 |
| Often or always |  | Never or rarely | Inadequate GWG | 2.03 (1.05 to 3.94) | 0.035 |  |
| Often or always |  | Never or rarely | Excess GWG | 1.88 (1.10 to 3.21) | 0.020 |  |
| **During the 3 months before pregnancy, did you want to eat when you were emotionally upset?** | 968 |  |  |  |  | 0.002 |
| Often or always |  | Never or rarely | Inadequate GWG | 0.88 (0.55 to 1.41) | 0.585 |  |
| Often or always |  | Never or rarely | Excess GWG | 1.60 (1.15 to 2.22) | 0.005 |  |
| **I eat sensibly when with others, but overeat when I’m alone** | 965 |  |  |  |  | <0.001 |
| Yes |  | No | Inadequate GWG | 1.54 (1.03 to 2.31) | 0.036 |  |
| Yes |  | No | Excess GWG | 1.88 (1.39 to 2.54) | <0.001 |  |
| **If others saw how much I ate, then I’d feel ashamed** | 964 |  |  |  |  | 0.005 |
| Yes |  | No | Inadequate GWG | 1.44 (0.87 to 2.39) | 0.160 |  |
| Yes |  | No | Excess GWG | 1.86 (1.27 to 2.72) | 0.001 |  |
| **When I am considering eating more than I should, or when I am considering eating a food that I feel is not particularly healthy, I tell myself it is okay because I will eat healthier later.** | 961 |  |  |  |  | 0.020 |
| Rarely, frequently, or always |  | Never | Inadequate GWG | 0.73 (0.47 to 1.14) | 0.165 |  |
| Rarely, frequently, or always |  | Never | Excess GWG | 1.29 (0.91 to 1.82) | 0.154 |  |
| **When I am considering eating more than I should, or when I am considering eating a food that I feel is not particularly healthy, I tell myself it is okay because I will compensate by eating less later.** |  |  |  |  |  | 0.754 |
| Rarely, frequently, or always | 959 | Never | Inadequate GWG | 0.95 (0.64 to 1.42) | 0.813 |  |
| Rarely, frequently, or always | 959 | Never | Excess GWG | 1.08 (0.80 to 1.45) | 0.610 |  |
| **When I am considering eating more than I should, or when I am considering eating a food that I feel is not particularly healthy, I tell myself it is okay because I will compensate by doing some exercise later.** | 962 |  |  |  |  | 0.107 |
| Rarely, frequently, or always |  | Never | Inadequate GWG | 0.98 (0.63 to 1.52) | 0.925 |  |
| Rarely, frequently, or always |  | Never | Excess GWG | 1.36 (0.98 to 1.89) | 0.069 |  |
| **I have a lot of fear regarding the health of my baby** | 950 |  |  |  |  | 0.080 |
| Yes |  | No | Inadequate GWG | 0.66 (0.40 to 1.09) | 0.107 |  |
| Yes |  | No | Excess GWG | 1.12 (0.80 to 1.57) | 0.509 |  |
| **Nausea related to pregnancy** | 967 |  |  |  |  | 0.833 |
| ≥1 time/day |  | Never or 1 time/week | Inadequate GWG | 0.89 (0.57 to 1.40) | 0.620 |  |
| ≥1 time/day |  | Never or 1 time/week | Excess GWG | 1.01 (0.72 to 1.41) | 0.954 |  |
| **Food cravings related to pregnancy** | 952 |  |  |  |  | 0.055 |
| ≥1 time/day |  | Never or 1 time/week | Inadequate GWG | 0.59 (0.38 to 0.92) | 0.019 |  |
| ≥1 time/day |  | Never or 1 time/week | Excess GWG | 0.92 (0.68 to 1.24) | 0.566 |  |
| **Eat something to cope with nausea** | 970 |  |  |  |  | 0.608 |
| Yes |  | No | Inadequate GWG | 0.87 (0.59 to 1.30) | 0.507 |  |
| Yes |  | No | Excess GWG | 0.87 (0.65 to 1.16) | 0.330 |  |
| **Avoiding eating to cope with nausea** | 970 |  |  |  |  | 0.359 |
| Yes |  | No | Inadequate GWG | 1.01 (0.63 to 1.63) | 0.959 |  |
| Yes |  | No | Excess GWG | 0.80 (0.56 to 1.14) | 0.217 |  |
| **Take medication to cope with nausea** | 970 |  |  |  |  | 0.174 |
| Use medications |  | None | Inadequate GWG | 0.39 (0.15 to 1.01) | 0.052 |  |
| Use medications |  | None | Excess GWG | 0.87 (0.49 to 1.56) | 0.649 |  |
| Other ways |  | None | Inadequate GWG | 0.81 (0.49 to 1.33) | 0.405 |  |
| Other ways |  | None | Excess GWG | 0.76 (0.52 to 1.11) | 0.151 |  |
| **Eat what I crave to cope with cravings** | 970 |  |  |  |  | 0.657 |
| Yes |  | Eat what I crave, no | Inadequate GWG | 0.83 (0.54 to 1.26) | 0.377 |  |
| Yes |  | Eat what I crave, no | Excess GWG | 0.96 (0.70 to 1.32) | 0.821 |  |
| **Avoid what I crave to cope with cravings** | 970 |  |  |  |  | 0.980 |
| Yes |  | Avoid what I crave, no | Inadequate GWG | 0.95 (0.52 to 1.72) | 0.865 |  |
| Yes |  | Avoid what I crave, no | Excess GWG | 0.96 (0.62 to 1.48) | 0.861 |  |
| **Distract myself to cope with cravings** | 970 |  |  |  |  | 0.775 |
| Yes |  | Distract myself, no | Inadequate GWG | 0.89 (0.53 to 1.48) | 0.642 |  |
| Yes |  | Distract myself, no | Excess GWG | 1.05 (0.73 to 1.51) | 0.797 |  |
| **Sleep** | 961 |  |  |  |  | 0.626 |
| ≥ 8 hr/day |  | < 8 hr/day | Inadequate GWG | 1.12 (0.75 to 1.68) | 0.568 |  |
| ≥ 8 hr/day |  | < 8 hr/day | Excess GWG | 1.15 (0.86 to 1.54) | 0.337 |  |
| **Total physical activity** | 894 |  |  |  |  | 0.347 |
| Medium |  | Low | Inadequate GWG | 0.64 (0.38 to 1.06) | 0.082 |  |
| Medium |  | Low | Excess GWG | 1.03 (0.72 to 1.49) | 0.861 |  |
| High |  | Low | Inadequate GWG | 0.80 (0.49 to 1.30) | 0.365 |  |
| High |  | Low | Excess GWG | 0.95 (0.66 to 1.38) | 0.790 |  |
| **Preferred body size image before pregnancy** | 965 |  |  |  |  | 0.014 |
| Underweight |  | Normal weight | Inadequate GWG | 1.47 (0.92 to 2.37) | 0.109 |  |
| Underweight |  | Normal weight | Excess GWG | 0.68 (0.46 to 1.01) | 0.054 |  |
| Overweight or obese |  | Normal weight | Inadequate GWG | 0.93 (0.42 to 2.05) | 0.856 |  |
| Overweight or obese |  | Normal weight | Excess GWG | 0.87 (0.50 to 1.53) | 0.632 |  |
| **Comparison between perceived and preferred body size image** | 965 |  |  |  |  | 0.014 |
| Small |  | Satisfied | Inadequate GWG | 0.54 (0.20 to 1.42) | 0.212 |  |
| Small |  | Satisfied | Excess GWG | 0.66 (0.34 to 1.28) | 0.217 |  |
| Large |  | Satisfied | Inadequate GWG | 1.06 (0.69 to 1.60) | 0.801 |  |
| Large |  | Satisfied | Excess GWG | 1.50 (1.09 to 2.06) | 0.012 |  |
| **Comparison between BMI and perceived body size image** | 966 |  |  |  |  | <0.001 |
| Small |  | Satisfied | Inadequate GWG | 0.88 (0.32 to 2.39) | 0.801 |  |
| Small |  | Satisfied | Excess GWG | 0.66 (0.30 to 1.46) | 0.309 |  |
| Large |  | Satisfied | Inadequate GWG | 1.20 (0.78 to 1.82) | 0.407 |  |
| Large |  | Satisfied | Excess GWG | 1.93 (1.42 to 2.64) | <0.001 |  |
| **Sitting time/day, hr** | 894 |  |  |  |  | 0.741 |
| Continuous |  | Continuous | Inadequate GWG | 0.99 (0.93 to 1.06) | 0.800 |  |
| Continuous |  | Continuous | Excess GWG | 1.01 (0.97 to 1.06) | 0.601 |  |
| **TPB score** | 963 |  |  |  |  | 0.941 |
| Continuous |  | Continuous | Inadequate GWG | 0.97 (0.80 to 1.19) | 0.800 |  |
| Continuous |  | Continuous | Excess GWG | 0.98 (0.84 to 1.13) | 0.738 |  |
| **Personality- Extraversion** | 952 |  |  |  |  | 0.690 |
| Continuous |  | Continuous | Inadequate GWG | 1.06 (0.92 to 1.21) | 0.430 |  |
| Continuous |  | Continuous | Excess GWG | 1.03 (0.94 to 1.14) | 0.496 |  |
| **Personality- Agreeableness** | 945 |  |  |  |  | 0.025 |
| Continuous |  | Continuous | Inadequate GWG | 0.82 (0.67 to 1.01) | 0.058 |  |
| Continuous |  | Continuous | Excess GWG | 1.06 (0.91 to 1.23) | 0.429 |  |
| **Personality- Conscientiousness** | 951 |  |  |  |  | 0.065 |
| Continuous |  | Continuous | Inadequate GWG | 1.01 (0.83 to 1.24) | 0.918 |  |
| Continuous |  | Continuous | Excess GWG | 0.86 (0.75 to 1.00) | 0.046 |  |
| **Personality- Emotional Stability** | 953 |  |  |  |  | 0.467 |
| Continuous |  | Continuous | Inadequate GWG | 0.92 (0.79 to 1.07) | 0.284 |  |
| Continuous |  | Continuous | Excess GWG | 0.94 (0.84 to 1.05) | 0.291 |  |
| **Personality- Openness** | 956 |  |  |  |  | 0.987 |
| Continuous |  | Continuous | Inadequate GWG | 0.99 (0.82 to 1.18) | 0.884 |  |
| Continuous |  | Continuous | Excess GWG | 1.00 (0.87 to 1.14) | 0.992 |  |
| The descriptive and univariable multinomial logistic regression analyses were complete case analyses.  CI, confidence interval; OR, odds ratio. | | | | | | |
